# Organ-specific transcriptional regulation by HFR1 and HY5 in response to shade in Arabidopsis

**DOI:** 10.3389/fpls.2024.1430639

**Published:** 2024-07-31

**Authors:** Ian Kin Yuen Choi, Amit Kumar Chaturvedi, Benny Jian Rong Sng, Kien Van Vu, In-Cheol Jang

**Affiliations:** ^1^ Temasek Life Sciences Laboratory, National University of Singapore, Singapore, Singapore; ^2^ Department of Biological Sciences, National University of Singapore, Singapore, Singapore

**Keywords:** cell elongation, HFR1, HY5, hypocotyl, transcription factor, shade avoidance response

## Abstract

Light is crucial for plants and serves as a signal for modulating their growth. Under shade, where red to far-red light ratio is low, plants exhibit shade avoidance responses (SAR). *LONG HYPOCOTYL IN FAR-RED 1* (*HFR1*) and *ELONGATED HYPOCOTYL 5* (*HY5*) are known to be negative regulators of SAR and physically interact with one another. However, transcriptional regulatory network underlying SAR by these two transcription factors has not been explored. Here, we performed organ-specific transcriptome analyses of *Arabidopsis thaliana hfr1-5*, *hy5-215* and *hfr1hy5* to identify genes that are co-regulated by HFR1 and HY5 in hypocotyls and cotyledons. Genes co-regulated by HFR1 and HY5 were enriched in various processes related to cell wall modification and chlorophyll biosynthesis in hypocotyls. Phytohormone (abscisic acid and jasmonic acid) and light responses were significantly regulated by HFR1 and HY5 in both organs, though it is more prominent under shade in cotyledons. HFR1 and HY5 also differentially regulate the expression of the cell wall-related genes for xyloglucan endotransglucosylase/hydrolase, expansin, arabinogalactan protein and class III peroxidase depending on the organs. Furthermore, HFR1 and HY5 cooperatively regulated hypocotyl responsiveness to shade through auxin metabolism. Together, our study illustrates the importance of the HFR1-HY5 module in regulating organ-specific shade responses in Arabidopsis.

## 1 Introduction

As sessile organisms, plants are subjected to a wide range of complex light conditions depending on their growth environment. Higher plants have acquired the ability to sense and decipher various light signals to control their development. In natural environments, plants acquire a set of responses to escape high density plant environment termed the shade avoidance response (SAR; Casal and Fankhauser, 2023). The SAR is prevalent in vegetative shade when plants are grown in proximity or in canopy shade, where available light is either reflected or transmitted by neighboring plant leaves, which resulted in phenotypic changes that include hyponasty, petiole elongation, hypocotyl elongation and early flowering (Casal, 2013; Roig-Villanova and Martínez-García, 2016; Casal and Fankhauser, 2023). The absorption spectra of photosynthetic pigments encompass the red (R) spectrum of the photosynthetically active radiation (PAR, 400-700 nm) and thus reflected or transmitted light by leaves are enriched in green or far-red (FR), resulting in a low R to FR light ratio (R:FR<1; Casal, 2013; Sessa et al., 2018). The detection of the FR-enriched light by R or FR light-absorbing phytochromes (phy) initiates SAR to achieve growth space and light quality or quantity at the expense of other developmental processes or defense budget (Casal, 2013; Pierik and Ballaré, 2021).

The primary organ for shade perception is known to be the cotyledons, which generate auxin in low R/FR to trigger hypocotyl elongation (Tanaka et al., 2002; Procko et al., 2014). However, expression of auxin responsive genes in the hypocotyls was found to be altered within 15 min shade exposure (Kohnen et al., 2016). In addition, localized auxin metabolism can also influence hypocotyl elongation in shade and high temperature independent of the cotyledons (Zheng et al., 2016), supporting organ-specific sensing of environmental cues to adjust their developmental program. Controlling shade avoidance downstream of the phytochromes is the PHYTOCHROME INTERACTING FACTOR-LONG HYPOCOTYL IN FAR-RED 1 (PIF-HFR1) module, where PIFs serve as positive regulators of shade avoidance (Hornitschek et al., 2012; Paulišić et al., 2021). On the other hand, HFR1 is mainly known to function through inhibitory interactions with the PIF family (Sessa et al., 2005; Lorrain et al., 2008). HFR1 has been shown to physically interact with another positive regulator of photomorphogenesis, ELONGATED HYPOCOTYL 5 (HY5; Jang et al., 2013), which suggests that they can also function under a shared signaling pathway. On the other hand, HY5 acts as a central regulator of light signaling by promoting photomorphogenic development through direct and/or indirect regulation of many light-responsive genes as well as auxin-responsive genes (Wang et al., 2021; Xiao et al., 2022). HY5 has been shown to regulate thermomorphogenesis in an organ-specific manner (Lee et al., 2021). Although both HFR1 and HY5 function as positive regulators of light signaling, they do not appear to share similar downstream regulation, besides displaying antagonistic roles with PIFs (Leivar and Monte, 2014; Pham et al., 2018). In addition, additive phenotypic changes observed in *hy5hfr1* under FR light suggest that they function in largely independent pathways (Kim et al., 2002; Jang et al., 2013).

Here, we identified the shared pathways co-regulated by HFR1 and HY5 through transcriptomic analysis in different organs (cotyledons and hypocotyls) of Col-0, *hfr1-5*, *hy5-215* and *hfr1hy5* seedlings under light or shade treatment. We found that HFR1 and HY5 regulate cell wall-related processes specifically in hypocotyls, while also involved in other light and hormonal responses in both organs. Moreover, HFR1 and HY5 also cooperatively influence the local auxin levels in each organ through regulation of auxin biosynthesis and auxin conjugation. Taken together, our results suggest that HFR1 and HY5 differentially regulate individual organs to modulate cell elongation and other developmental processes such as biotic defense and chlorophyll biosynthesis under shade.

## 2 Materials and methods

### 2.1 Plant materials and growth conditions

All Arabidopsis plants used in this study were of the Col-0 ecotype. *hfr1-5* (Sessa et al., 2005) and *hy5-215* (Oyama et al., 1997) were previously described. *hfr1hy5* was generated through crossing of *hfr1-5* and *hy5-*215. Seeds were surface-sterilized using 20% bleach with 0.01% Tween-20 for 10 min, before rinsing five times with sterile water. The sterilized seeds were sown on half-strength Murashige and Skoog (MS) medium containing 0.8% agar, 1% sucrose, and 0.05% 2-(*N*-morpholino)-ethanesulfonic acid (MES), at pH 5.7 and cold-stratified at 4°C for 3 d in darkness. The seeds were then transferred to long day conditions (16 h light; 8 h dark cycle), under 100 μmol m^-2^ s^-1^ white light at 22°C and 60% relative humidity for 9 d, unless stated otherwise. For monochromatic light treatment, cold-stratified seeds were grown under different R (10, 20, 40, 80 µmol m^-2^ s^-1^), FR (1, 2, 5, 10 µmol m^-2^ s^-1^) and blue (B; 10, 20, 40, 80 µmol m^-2^ s^-1^) light for 4 d. For shade treatment, stratified seeds were grown under control white light (100 μmol m^-2^ s^-1^, R:FR=3.0) for 4 d before transferring to shade condition (20 μmol m^-2^ s^-1^, R:FR=0.2) for 5 d. For auxin biosynthesis inhibition, cold-stratified seeds were grown in ½ MS agar supplemented with 5 µM L-kynurenine (kyn; Sigma-Aldrich), 25 µM yucasin (Santa Cruz Biotechnology) or both kyn and yucasin, under control white light (100 μmol m^-2^ s^-1^, R:FR=3.0) for 9 d or under shade treatment.

### 2.2 Phenotypic measurement

Photographs of the plant samples were documented after each treatment. For cotyledon area, cotyledons were dissected and placed on 1% agarose gel with the adaxial side facing upwards before photographs were captured. Images of hypocotyl epidermal cells were taken using scanning electron microscope (SEM) JSM-6360LV (JEOL). Hypocotyl length, hypocotyl epidermal cell length and cotyledon area were subsequently measured using ImageJ software (Schindelin et al., 2012).

### 2.3 Genomic DNA extraction and genotyping of *hfr1hy5* double mutant

Genomic DNA extraction was performed to confirm the *hfr1-5*, *hy5-215* and *hfr1hy5* backgrounds through genotyping PCR. Plant tissues (1-2 leaves) from each genotype were harvested and homogenized in 400 μl DNA extraction buffer (0.2 M Tris, pH 7.5; 25 mM EDTA; 0.25 M NaCl and 0.5% SDS (w/v)) using a micropestle. The samples were centrifuged for 5 min at 13,000 g to pellet the cell debris. 350 μl of supernatant was mixed with equal volume of isopropanol for DNA precipitation before centrifugation at 13,000 g for 10 min. The DNA pellet was washed with 70% ethanol, followed by an additional 5 min centrifugation before the supernatant was decanted. The pellet was air dried to remove any ethanol. The resulting DNA pellet was subsequently dissolved in 50 μl of sterile water. For genotyping PCR, primers used were listed in **Supplementary Table 1**. For *hfr1-5*, the primers were obtained using the online tool (http://signal.salk.edu/tdnaprimers.2.html). The target sequences in the extracted genomic DNA were amplified using the designed primers. The amplified PCR products were run in gel electrophoresis to analyze the product size.

### 2.4 RNA sequencing data analysis

For transcriptome sequencing, 4-d-old Col-0, *hfr1-5*, *hy5-215* and *hfr1hy5* seedlings grown in long day condition with or without additional treatment with shade for 1 h was used. Untreated- and shade-treated seedlings were transferred into RNAlater solution (Thermo Scientific) containing 200 μM actinomycin D (Sigma-Aldrich) and vacuumed for 10 min. The seedlings were dissected to harvest the cotyledons and hypocotyls. The RNA was extracted from organ samples using Ribospin™ Plant RNA extraction kit (GeneAll). The quality of the extracted RNA was measured using the NanoDrop™ 1000 spectrophotometer (Thermo Fisher Scientific), ensuring that the samples had a 260/280 ratio of > 1.8 and a 260/230 ratio of > 2.2. RNA-Seq was performed using NovaSeq 6000 system (Macrogen). The raw reads were refined by trimming the adapter sequences and removing low-quality reads before subsequent alignment and mapping to Arabidopsis TAIR10 reference genome. Gene expression was determined using TMM normalization, which was performed using edgeR (version 3.22.5; R version 3.5.0). The RNA-seq was performed on three biological replicates.

### 2.5 Differentially expressed genes analysis

DEGs were selected based on the conditions of log_2_ fold change ≥ 1 or ≤ -1 and *P*-value < 0.05. Principal component analysis (PCA) and K-means clustering of DEGs were performed using MATLAB R2020a. The Venn diagrams were generated using the Interactivenn web tool (Heberle et al., 2015; http://www.interactivenn.net). Gene ontology (GO) enrichment was analyzed using the Database for Annotation, Visualization and Integrated Discovery (DAVID version 6.8; Huang et al., 2009; https://david.ncifcrf.gov/), while the GO dotplot was created with the R software (version 4.1.1), using the ggplot2 package (https://www.rdocumentation.org/packages/ggplot2/versions/3.3.5). Lastly, the hierarchical cluster heatmaps were generated using ClustVis web tool (Metsalu and Vilo, 2015; https://biit.cs.ut.ee/clustvis/).

### 2.6 Quantitative reverse transcription-polymerase chain reaction

For gene expression analysis, a minimum of 20 seedlings were harvested per sample and pooled together before being frozen, ground and stored immediately in liquid nitrogen. Total RNA was extracted using the Ribospin™ Plant RNA extraction kit (GeneAll). The cDNA synthesis was performed using qScript cDNA Synthesis Kit (Quantabio). For each reaction, 1 μg of RNA was added to the reaction mix for a total volume of 20 μl. After reverse transcription reaction, the synthesized cDNA was diluted 10 times with nuclease-free water and stored at -20°C. qRT-PCR was performed using Takara SYBR Premix Ex Taq (Takara Bio) on the Biorad CFX connectTM real-time system (Bio-Rad Laboratories). Relative gene expressions quantified by qRT-PCR were carried out in triplicates. *UBIQUITIN 11* (*UBQ11*) was used as internal reference. Primers used for gene expression analysis were listed in **Supplementary Table 1**.

### 2.7 Quantification of chlorophyll and carotenoid content

Total chlorophyll and carotenoid content were extracted from 7-d-old Col-0, *hfr1-5*, *hy5-21*5 and *hfr1hy5* seedlings that were subjected to 0 h and 1 h shade treatment. The seedlings were harvested and ground in liquid nitrogen using mortar and pestle. The chlorophyll content was extracted from the ground samples using 95% ethanol. The samples were then incubated overnight in darkness at 4°C. Subsequently, the samples were centrifuged at 13,000 g for 10 min, 4°C. The supernatant was loaded into 96-well microplates and the absorbance was measured at 470 nm, 648 nm and 664 nm using Spark^®^ multimode microplate reader (Tecan). The total chlorophyll content, comprised of the sum of chlorophyll a (Chl *a*) and chlorophyll b (Chl *b*), and carotenoid content (C) were calculated with the following formulae (Lichtenthaler, 1987): Chl *a*= 13.36 × A_664_ – 5.19 × A_648_, Chl *b* = 27.43 × A_648_ – 8.12 × A_664_, C= (1000 × A_470_ − 2.13Chl *a* − 97.64Chl *b*)/209.

### 2.8 Quantification of phytohormone levels

For quantification of indole-3-acetic acid (IAA), jasmonic acid (JA) and abscisic acid (ABA), 7-d-old Col-0, *hfr1-5*, *hy5-21*5 and *hfr1hy5* seedlings were subjected to 0 h and 1 h shade treatment. Seedlings were dissected into cotyledon and hypocotyl samples that were separately collected, weighed, and frozen using liquid nitrogen. Phytohormones were extracted by adding 80% methanol to 100 mg of ground tissue samples. The samples were shaken on ice for 30 min, followed by centrifugation at 13,000 g, 4°C for 5 min. The supernatant was transferred to a new 1.5 ml tube before drying in the centrifuge concentrator, Concentrator plus (Eppendorf). After drying, 100 µl of 100% methanol was added to each sample and vortexed to homogenize samples. The samples were then centrifuged at 13,000 g, 4°C for 10 min to pellet any precipitates. The phytohormone levels in the extracts were quantified using ultra-high performance liquid chromatography tandem mass spectrometry (UHPLC-MS/MS), consisting of 1290 Infinity II LC System (Agilent, USA) connected to the Agilent 6495 Triple Quadrupole LC/MS system (Agilent, USA). For separation of analytes, the Accucore™ RP-MS column (Thermo Fisher Scientific, USA) was used. Mobile phase A (5% (v/v) acetonitrile, 5 mM acetic acid) and mobile phase B (95% (v/v) acetonitrile, 5 mM acetic acid) were employed for elution. The elution profile was set as follows: 0–3 min, 5% B; 3–6 min, 5–95% B; 6–10 min, 95%; 10–10.1 min, 95–5% B; and 10.1–11 min, 5% B. The mobile phase flow rate was set at 0.3 ml min^-1^ with a 5 µl injection volume. Electrospray ionization was operated in negative ion mode. The concentrations of extracts were normalized to the fresh weight. IAA and ABA standards were procured from Sigma-Aldrich. JA standard was obtained from Olchemim.

### 2.9 Statistical analysis

All experiments were performed with three independent replicates. Levene’s test was used to confirm equal variance across sample groups. The Welch’s *t*-test was applied to investigate the significant differences between samples with regards to hypocotyl length, cell length, cell number and cotyledon area. Student’s *t*-test was employed to determine statistical differences in carotenoid, chlorophyll and phytohormone content.

## 3 Results

### 3.1 *hfr1hy5* double mutant displays additive shade avoidance phenotypes

The *hy5-1hfr1-201* double mutant was previously generated to explore the genetic interaction between *HFR1* and *HY5* (Kim et al., 2002). However, *hy5-1* and *hfr1-201* were of different ecotypes (*hy5-1*, Landsberg *erecta*; *hfr1-201*, Columbia; Oyama et al., 1997; Kim et al., 2002). We generated a *hfr1hy5* double mutant with the same ecotype (Col-0), through the crossing of *hfr1-5* with *hy5-215* (**Supplementary Figure 1**; Oyama et al., 1997; Sessa et al., 2005). Then, we performed phenotypic analysis of *hfr1hy5* under R and B light in addition to FR light tested elsewhere (**Supplementary Figure 2**; Kim et al., 2002; Jang et al., 2013). Under all three monochromatic light conditions, *hfr1hy5* displayed longer hypocotyls compared to those of *hfr1-5* or *hy5-215*, which was also consistently observed across different light intensities (**Supplementary Figure 2**). This additive hypocotyl elongation phenotype of *hfr1hy5* under different wavelengths of light suggests a subtle regulation by HFR1 and HY5 on overall growth and development of Arabidopsis plants in natural light environments.

**Figure 1:**
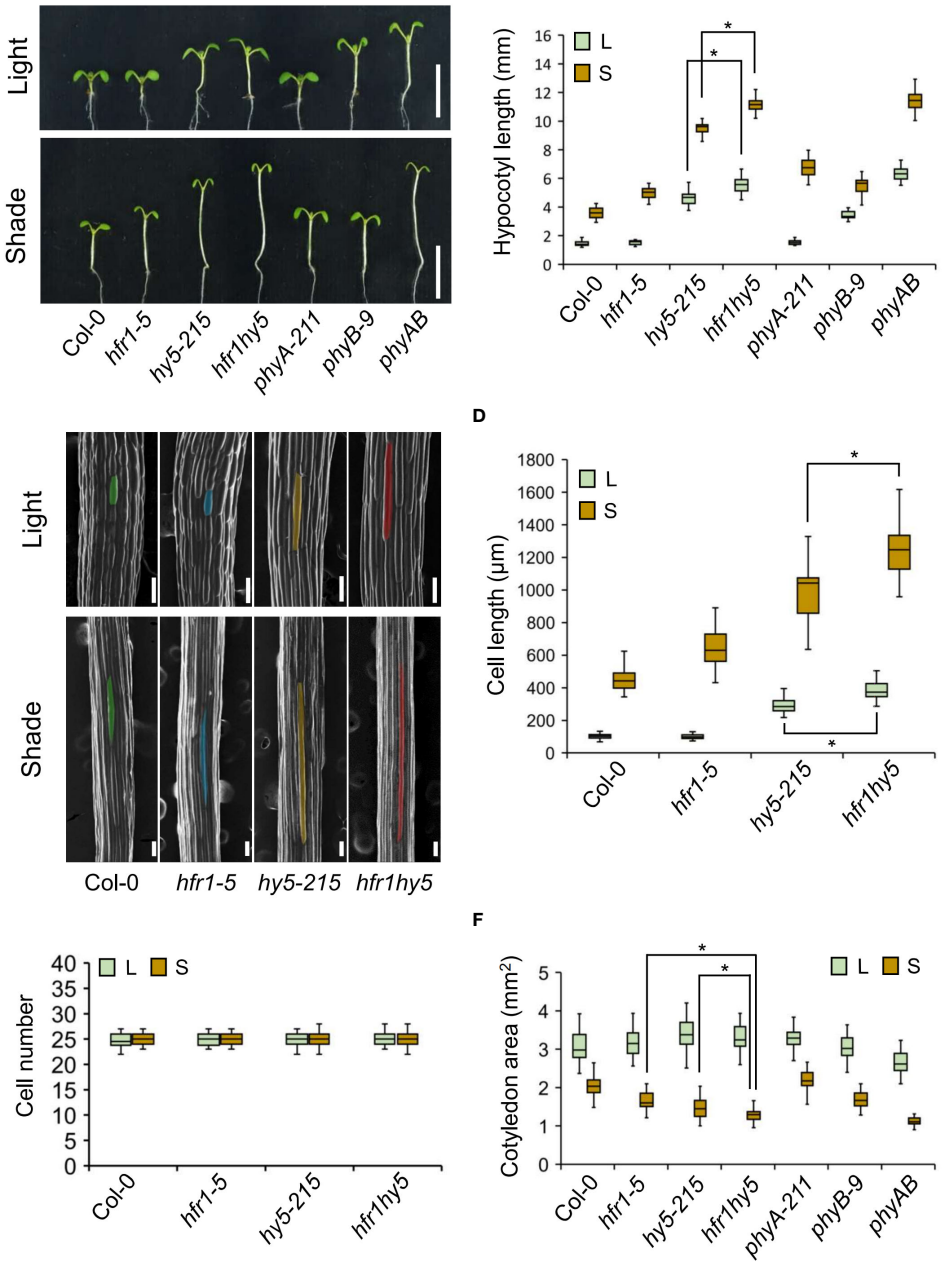
Shade-induced phenotypes of Arabidopsis mutants, *hfr1-5, hy5-215* and *hfr1hy5*. **(A)** Seedling phenotype of Col-0, *hfr1-5, hy5-215*, *hfr1hy5*, *phyA-211*, *phyB-9* and *phyAB* mutants grown under light and shade. Scale bar, 5 mm. Seeds of indicated lines were planted on ½ MS supplemented with 1% sucrose and grown under white light for 4 days before they were kept in white light or transferred to shade condition for additional 5 days. **(B)** Hypocotyl length of seedlings shown in **(A)**, light condition (L), shade condition (S), n=30. **(C)** SEM images of hypocotyl epidermal cells from Col-0, *hfr1-5*, *hy5-215* and *hfr1hy5*. Colored areas denote the individual cells used for cell length measurements. Scale bar, 100 µm. **(D)** Hypocotyl epidermal cell length (n=50) and **(E)** cell number (n=15) of seedlings shown in **(C)**. **(F)** Cotyledon area of seedlings shown in **(A)**, n=60. All results were obtained from the average of three independent replicates. The boxes in each graph extend from the 25th to 75th percentiles and the line in the middle of the box is plotted at the median. Whiskers represent the 1.5× interquartile range. n represents the sample size per independent replicate. Welch’s *t*-test was used to determine statistical difference between two samples; *P < 0.05.

Hence, to observe the genetic interaction of HFR1 and HY5 under natural environmental conditions, the seedlings of *hfr1hy5* were subjected to shade treatment. Under normal light condition, *hy5-215* displayed longer hypocotyls while *hfr1-5* shared similar hypocotyl length when compared to Col-0 (**Figures 1A, B**). However, *hfr1hy5* had longer hypocotyls compared to *hy5-215* (**Figures 1A, B**). Under shade, *hfr1hy5* similarly displayed additive phenotypes in terms of increased hypocotyl elongation compared to the single *hfr1-5* and *hy5-215* mutants (**Figures 1A, B**). Interestingly, the hypocotyl length of *hfr1hy5* was longer than that of *phyA* and *phyB* mutants under shade and its phenotype was comparable to *phyAB* double mutant (**Figures 1A, B**). HFR1 and HY5 did not influence the *phyA* and *phyB* expressions under both light and shade (**Supplementary Figure 3**), which implies the importance of HFR1 and HY5 in downstream signaling pathways. In addition, the increase in hypocotyl length under shade observed in *hfr1hy5* was 2.5-fold higher than the hypocotyl elongation observed in Col-0 (**Supplementary Figure 4A**), supporting the crucial roles of HFR1 and HY5 in shade. The morphology of the hypocotyl epidermal cells was also examined for differences in the hypocotyl elongation (**Figures 1C, D**). As reflected in the hypocotyl lengths, the cell length in *hfr1-5* hypocotyls was similar to that in Col-0 under light (**Figures 1C, D**). Hypocotyl cells of *hy5-215* and *hfr1hy5* were longer than those of Col-0 or *hfr1-5* (**Figures 1C, D**). *hfr1hy5* displayed longer cells than the single mutants under both conditions, though there were no significant differences in the cell number (**Figures 1C–E**). This indicates that the longer hypocotyl in *hfr1hy5* seedlings resulted from an enhanced cell elongation rather than an increase in cell number. Additionally, reduced cotyledon area was observed in *hfr1hy5* compared to the single *hfr1* and *hy5* mutants under shade (**Figure 1F**). Other parameters such as chlorophyll and carotenoid content were lower in *hfr1hy5* compared to *hfr1-5* and *hy5-215* for both light and shade conditions (**Supplementary Figures 4B, C**).

### 3.2 Transcriptome analysis reveals differential regulation by HFR1 and HY5 in cotyledon and hypocotyl of *Arabidopsis thaliana*

**Figure 2:**
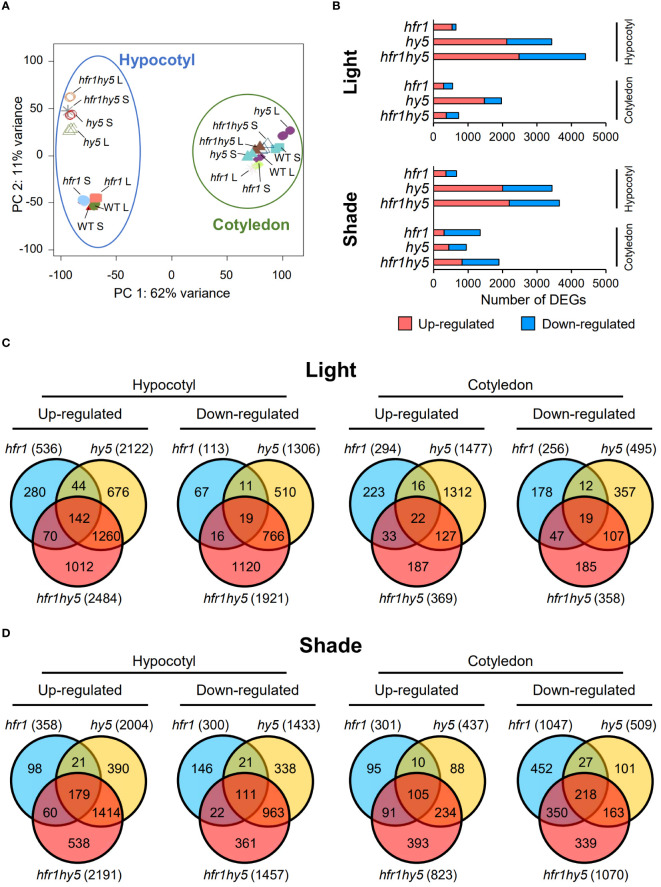
DEGs analysis showing organ-specific differences of genotypes under shade. **(A)** PCA analysis of organ-specific transcriptomes from Col-0 (wild-type, WT), *hfr1*-5 (*hfr1*), *hy5-215* (*hy5*) and *hfr1hy5* that were grown under light (L) or transferred to shade for 1 h (S). Data points within the blue circle and green circle represent hypocotyl and cotyledon samples, respectively. **(B)** Distribution of DEGs in *hfr1*, *hy5* and *hfr1hy5* hypocotyl and cotyledon samples treated under light (Light) and 1 h shade (Shade). DEGs obtained from each genotype were plotted separately as up-regulated (pink, adjusted *P* ≤ 0.05 and log_2_fold change ≥ 1), or down-regulated (blue, adjusted *P* ≤ 0.05 and log_2_fold change ≤ -1). Venn diagrams of up- and down-regulated DEGs from **(C)** light- and **(D)** 1 h shade-treated *hfr1*, *hy5* and *hfr1hy5* hypocotyls in comparison to Col-0. The values in each Venn diagram represent the number of DEGs that each segment constitutes.

Although the additive SAR phenotypes observed in *hfr1hy5* suggest that HFR1 and HY5 have distinctive roles in regulating SAR, HFR1 and HY5 interaction has been demonstrated through *in vitro* pull-down assay and *in vivo* co-immunoprecipitation (Jang et al., 2013). This supports the notion that HFR1 and HY5 could co-regulate a plethora of genes through their physical interaction as a module. In addition, the phenotypic differences observed in cotyledon and hypocotyl prompted us to explore organ-specific transcriptional regulation of SAR by HFR1 and HY5. Hence, we conducted RNA-sequencing (RNA-seq) for cotyledon and hypocotyl samples of Col-0, *hfr1-5* (*hfr1*)*, hy5-215* (*hy5*) and *hfr1hy5* under light and shade treatment for 1 h. Principal component analysis (PCA) of the transcriptomes showed that the cotyledon samples were clustered away from the hypocotyl samples, suggesting large differences between the cotyledon and hypocotyl transcriptome (**Figure 2A**). In addition, the cotyledon samples from different genotypes and treatments were more tightly clustered compared to the spread-out hypocotyl samples (**Figure 2A**). In particular, the *hy5* and *hfr1hy5* hypocotyls were distanced from the Col-0 and *hfr1* hypocotyls (**Figure 2A**). This implies that there was a larger transcriptome difference among the genotypes in hypocotyls compared to cotyledons. Due to differences observed across the genotypes in different organs, we examined the influence of HFR1 and HY5 on their individual expressions in specific organs. *HFR1* expression was induced under shade in both cotyledons and hypocotyls but expression was reduced for both light and shade treatment in *hy5-215* compared to Col-0 (**Supplementary Figure 5**). In contrast, *HY5* expression was reduced under shade and further reduced in *hfr1-5* for cotyledons though differences in expression were not significant in hypocotyls (**Supplementary Figure 5**). This suggests that HFR1 and HY5 collectively promote their transcription though the influence of HFR1 on *HY5* expression is more specific to cotyledons.

To explore the processes regulated by HFR1 and HY5, DEGs from genotypes *hfr1-5, hy5-215* and *hfr1hy5* were identified, using Col-0 as the control for light treatment comparison (Light, **Figure 2B**) and for shade treatment comparison in cotyledons and hypocotyls (Shade, **Figure 2B**). The DEGs were filtered with a cutoff of log_2_-fold change >1 or <−1 for up- or down-regulated genes with *P*-value <0.05. DEG analysis showed that the number of up- and down-regulated DEGs in light-treated hypocotyls were higher in *hy5* and *hfr1hy5* compared to *hfr1*, with *hfr1hy5* having the highest number of DEGs (*hfr1*<*hy5*<*hfr1hy5*; Hypocotyl and Light, **Figure 2B**). This corresponds with hypocotyl lengths observed in the *hfr1-5*, *hy5-215* and *hfr1hy5* (**Figure 1**). In shade-treated hypocotyl DEGs, a similar trend was also observed compared to light-treated hypocotyls (Hypocotyl and Shade, **Figure 2B**). In comparison, there were no distinct patterns observed between the genotypes in either the light or shade comparisons for the cotyledons, although *hy5* cotyledons under light has a higher number of DEGs compared to *hfr1* or *hfr1hy5* (Cotyledon and Light, **Figure 2B**). The similar patterns observed in light and shade DEGs (low number of DEGs in *hfr1* hypocotyl, high number of DEGs in *hy5* and *hfr1hy5*, and no clear pattern in cotyledons) suggest that the plant genotype is more important than the treatment in regulating the organ-specific transcriptomes. Additionally, shade-responsive genes were also identified in each genotype, using the respective light-treated samples as the control (**Supplementary Figure 6A**). Analysis of hypocotyl shade-responsive genes showed that Col-0 had the highest number of DEGs while *hfr1hy5* had the lowest (Col-0>*hfr1*>*hy5*>*hfr1hy5*; Hypocotyl, **Supplementary Figure 6A**). This suggests that many of the shade-responsive genes were already up-regulated under light in *hfr1hy5* hypocotyls. On the other hand, cotyledon shade-responsive DEGs were similar across the different genotypes (Cotyledon, **Supplementary Figure 6A**).

To understand the specific and shared function of HFR1 and HY5, DEGs up- or down-regulated under light and shade were analyzed using Venn diagrams (**Figures 2C, D**). Analysis of the DEGs in hypocotyls under light revealed that a large portion of DEGs were shared between *hy5* and *hfr1hy5*, with 36.2% (1260) being up-regulated DEGs, and 30.5% (766) being down-regulated DEGs. (**Figure 2C**, hypocotyl). This supports HY5 playing a larger role in the genetic interaction between HFR1 and HY5. Interestingly, DEGs specific to *hfr1hy5* constituted 1012 (29%) of the up-regulated DEGs and 1120 (44.6%) of the down-regulated DEGs in hypocotyls (Hypocotyl, **Figure 2C**). These DEGs specific to *hfr1hy5* could entail the interaction of HFR1 and HY5 and distinct additive regulation in hypocotyl under light (Light, **Figure 1B**). Unlike hypocotyls under light, the highest percentage of DEGs in cotyledons under light were specific to *hy5-215*, constituting 68.3% (1312) of up-regulated DEGs; and 39.4% (357) of the total up- and down-regulated cotyledon DEGs, respectively (Cotyledon, **Figure 2C**). *hfr1hy5*-specific DEGs represented only 9.7% (187) of the total up-regulated DEGs and 20.4% (185) of down-regulated DEGs under light. This could underlie the absence of additive regulation under light in the cotyledon area (Light, **Figure 1B**).

HFR1 and HY5 are positive regulators of photomorphogenesis, and it is evident from the number of DEGs up- or down-regulated under shade treatment (Hypocotyl, **Figure 2D**). The shared genes between *hy5* and *hfr1hy5* increased to 52% (1414) and 49.1% (963) of the total up- and down-regulated hypocotyl DEGs under shade respectively (Hypocotyl, **Figure 2D**). In shade-treated cotyledons, a similar increase in up-regulated DEGs was observed for *hy5* and *hfr1hy5* shared DEGs (23.0%, 234 DEGs; Cotyledon, **Figure 2D**). Shaded hypocotyls of *hfr1hy5* displayed a reduced number of DEGs, which made up 19.9% (538) and 18.4% (361) of the up- and down-regulated DEGs respectively (Hypocotyl, **Figure 2D**). In contrast, shaded cotyledons of *hfr1hy5* showed an increase of up-regulated DEGs to 38.0% (393). In addition, the number of down-regulated *hfr1*-specific DEGs also increased to 27.4% (452), as compared to other genotypes (Cotyledon, **Figure 2D**). This implies that HFR1 activity is more prominent under shade in cotyledons and supports the role of HFR1 as a negative regulator of SAR.

### 3.3 HFR1 and HY5 cooperatively regulate cell elongation and hormone responses in specific organs

**Figure 3:**
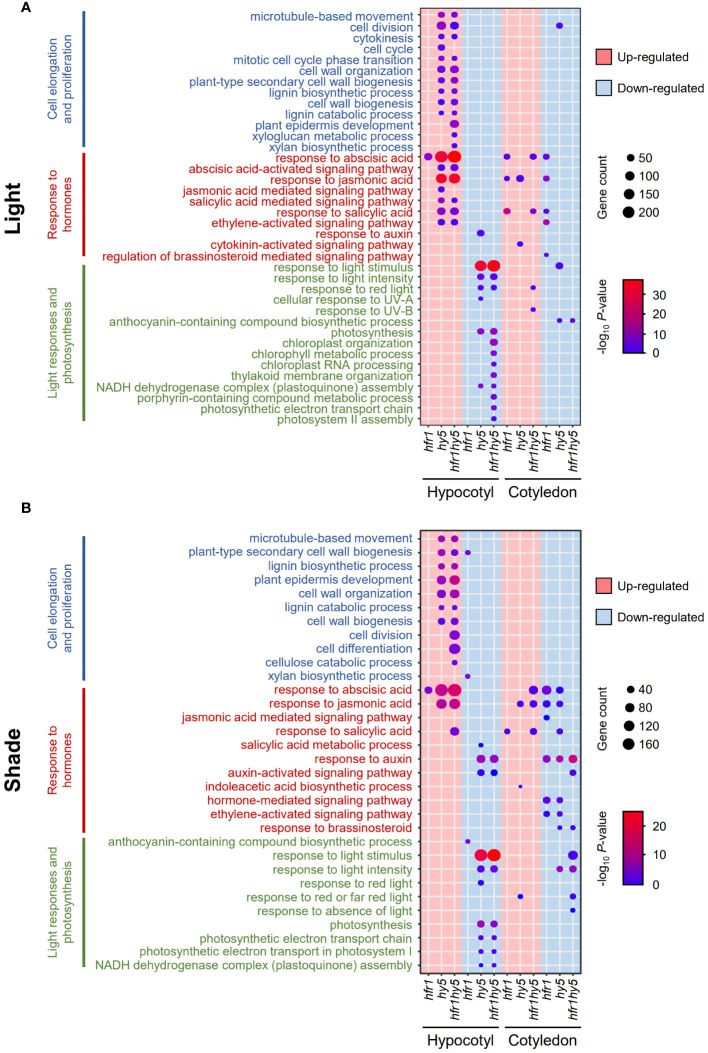
Identification of biological processes regulated by HFR1 and HY5 in hypocotyls and cotyledons under light and shade. Dot plot of GO terms from up- (pink) and down-regulated (blue) DEGs from **(A)** light- and **(B)** shade-treated *hfr1*, *hy5* and *hfr1hy5* DEGs in hypocotyls and cotyledons. DEGs were obtained through comparison with the respective Col-0 samples. Dot sizes correspond to the number of genes while the dot color represents the -log_10_ *P*-value.

To deduce the biological relevance of DEGs related to HFR1 and HY5, GO enrichment analysis was performed. Consistent with the hypocotyl phenotypes across the genotypes, GO terms related to cell proliferation and cell elongation were mainly found in *hy5* and *hfr1hy5* hypocotyls under light but not in *hfr1* hypocotyls (Hypocotyl, Pink, **Figure 3A**). Additionally, more cell wall-related GO terms such as ‘plant epidermis development’ and ‘xyloglucan metabolic process’ were observed in *hfr1hy5* hypocotyls compared to *hy5* hypocotyls (Hypocotyl, Pink, **Figure 3A**). GO terms related to hormones such as abscisic acid (ABA), jasmonic acid (JA), salicylic acid (SA) and ethylene were up-regulated in *hy5* and *hfr1hy5* hypocotyls under light (Hypocotyl, Pink, **Figure 3A**). This suggests the involvement of HFR1 and HY5 in modulating abiotic and biotic stresses. GO terms for light response and photosynthesis were down-regulated in *hfr1hy5* and *hy5* hypocotyls (Hypocotyl, Blue, **Figure 3A**), supporting the role of HY5 in regulating biosynthesis of photosynthetic pigments under light (Toledo-Ortiz et al., 2014). Moreover, GO terms related to chloroplast were overrepresented in *hfr1hy5* compared to *hy5* (Hypocotyl, Blue, **Figure 3A**), highlighting the role of HFR1 in chloroplast metabolism. In contrast, there were fewer GO terms enriched in the cotyledon DEGs under light (Cotyledon, **Figure 3A**). Only *hy5* down-regulated DEGs were enriched for the ‘cell division’ GO term for cell proliferation and cell elongation (Cotyledon, Blue, **Figure 3A**). Interestingly, only *hfr1* DEGs were down-regulated for hormone-related GO terms (Cotyledon, Blue, **Figure 3A**). *hy5* and *hfr1hy5* DEGs were also down-regulated for the GO term ‘anthocyanin-containing compound biosynthetic process’, supporting HY5 function in anthocyanin production (Cotyledon, Blue, **Figure 3A**).

Under shade, overall GO terms represented in both organs across genotypes were similar to those shown under light (**Figure 3B**). There was similarly higher representation of cell elongation and proliferation in up-regulated *hfr1hy5* DEGs compared to *hy5* DEGs in shade-treated hypocotyls (Hypocotyl, Pink, **Figure 3B**). Responses to ABA and JA were also up-regulated in both *hy5* and *hfr1hy5* hypocotyl DEGs under shade (Hypocotyl, Pink, **Figure 3B**). However, response to ABA was observed to be only up-regulated in *hfr1hy5* cotyledon DEGs while *hfr1* and *hy5* cotyledon DEGs were down-regulated for ABA and JA responses (Cotyledon, **Figure 3B**). In addition, ‘response to auxin’ was down-regulated in both *hy5* and *hfr1hy5* hypocotyls and cotyledons (Blue, **Figure 3B**). For GO terms related to light responses and photosynthesis, there were similar number of GO terms enriched in both *hy5* and *hfr1hy5* DEGs (Hypocotyl, Blue, **Figure 3B**).

Next, we analyzed the GO terms for each genotype in response to shade treatment. GO term related to ‘shade avoidance’ was only found in up-regulated hypocotyl DEGs (Hypocotyl, Pink, **Supplementary Figure 6B**). GO terms related to auxin signaling and response were enriched across all genotypes and organs under shade (Hypocotyl, Pink, **Supplementary Figure 6B**). While the response to auxin was still up-regulated under shade in *hy5* and *hfr1hy5* DEGs, the number of DEGs and statistical significance was reduced as compared to Col-0. In contrast, other phytohormone responses (ethylene, JA and ABA) were down-regulated in hypocotyls whereas up-regulated in cotyledons (**Supplementary Figure 6B**).

We further investigated the genotype-specific DEGs, which may further explain the individual phenotypes observed (**Supplementary Figure 7**). In hypocotyls, ABA response was up-regulated in *hfr1*-specific DEGs (**Supplementary Figure 7A**). For *hy5*-specific DEGs, defense responses were up-regulated as well as cell cycle-related GOs (**Supplementary Figure 7A**), which may contribute to the longer hypocotyls observed in *hy5-215* (**Figure 1**). Up-regulated DEGs in *hfr1hy5* were enriched in GOs mainly related to oxidative stress (**Supplementary Figure 7A**). On the other hand, light responses and chloroplast related GOs were downregulated in *hy5* and *hfr1hy5* hypocotyls (**Supplementary Figure 7A**), which could contribute to the reduced chlorophyll levels observed in *hy5-215* and *hfr1hy5* seedlings (**Supplementary Figure 4B**). In cotyledons, GOs were mainly enriched in *hfr1* for both up- and down-regulated DEGs, which were related to abiotic and biotic stress responses (**Supplementary Figure 7A**). This supports the importance of HFR1 even under optimal light conditions. However, under shade, only a few GOs were found to be enriched in the genotype-specific DEGs (**Supplementary Figure 7B**). In hypocotyls, up-regulated *hy5*-specific DEGs were enriched in ‘xylan biosynthetic process’ as well as ABA response (**Supplementary Figure 7B**). Similar to light condition, up-regulated *hfr1hy5*-specific DEGs were also enriched in GOs related to oxidative stress (**Supplementary Figure 7B**). As for cotyledons under shade, only down-regulated *hfr1*-specific DEGs were enriched for GOs such as ‘response to abscisic acid’ and ‘response to wounding’ (**Supplementary Figure 7B**).

As responses to JA and ABA were enriched especially in *hy5-215* and *hfr1hy5* hypocotyls under both light and shade, the JA and ABA levels were quantified to determine if the JA and ABA responses resulted from differences in phytohormone levels (**Supplementary Figure 8**). We found that both JA and ABA levels were relatively low in *hy5-215* and *hfr1hy5* hypocotyls compared to Col-0 and *hfr1-5* hypocotyls (**Supplementary Figure 8**). This result might be correlated with enriched GO terms ‘response to JA’ and ‘response to ABA’ in *hy5* and *hfr1hy5* hypocotyls (**Figure 3**). Taken together, HFR1 and HY5 differentially regulate many biological processes driving plant growth, light signaling, and stress responses in an organ-specific manner.

### 3.4 HFR1-HY5 module regulates cell expansion as well as abiotic and biotic stresses under shade

**Figure 4:**
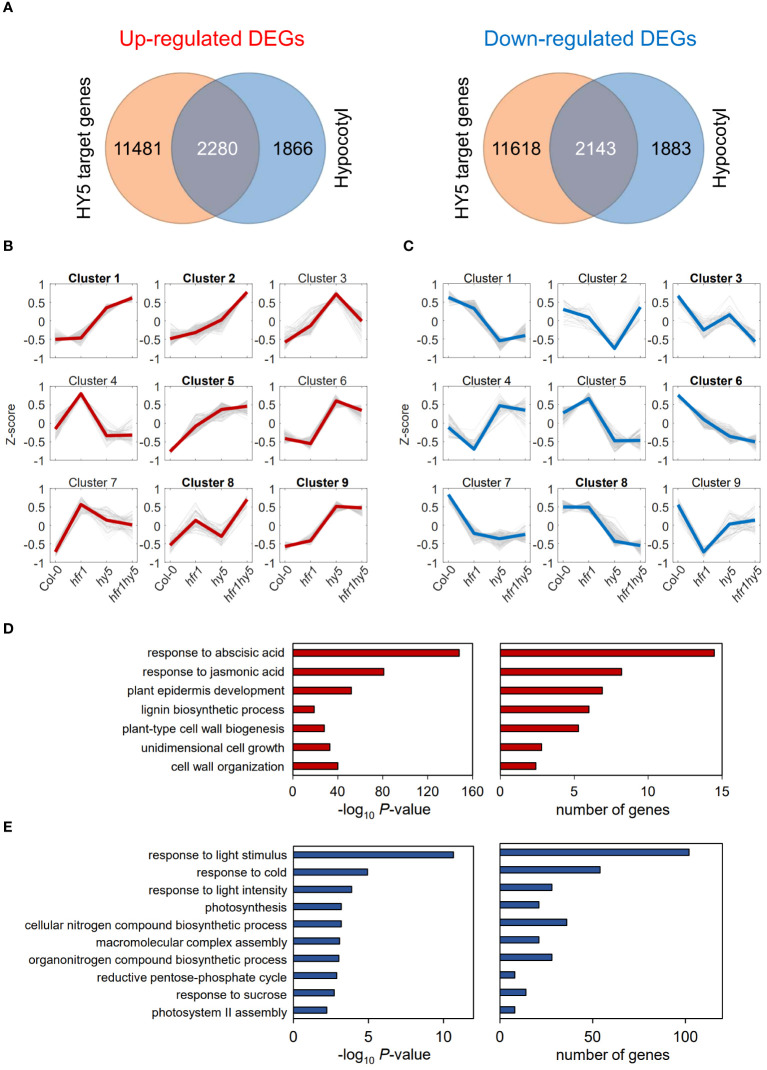
Identification of biological processes directly regulated by HFR1 and HY5 in hypocotyls under shade. **(A)** Venn diagrams display the overlap between HY5-target genes obtained from HY5 ChIP-seq data (Burko et al., 2020) and the up- (left) or down-regulated (right) DEGs found in the hypocotyls. Overlapped DEGs that were **(B)** up- or **(C)** down-regulated under shade were categorized into nine different clusters through K-means clustering. Expression changes in y-axis represent Z-score of log_2_-fold change of the DEGs. The thin grey lines represent the individual genes in each cluster. The thick lines (red or blue) represent the centroids of each cluster. **(D)** Enriched GO terms from clusters labelled in bold shown in **(B)**. **(E)** Enriched GO terms from clusters labelled in bold shown in **(C)**.

Previous studies have shown that HFR1 regulates auxin and ethylene-mediated growth, as well as biotic stress resistance under shade (Sng et al., 2023). While HFR1 does not have a DNA-binding domain (Fairchild et al., 2000), HY5 binds to the “ACGTG” consensus sequence in the promoter region of its target genes (Lee et al., 2007). Hence, the interaction of HFR1 with HY5 may serve as an additional layer of transcriptional regulation for HY5-target genes. Looking at the co-regulated genes and oppositely-regulated genes, 16% (42) of the total DEGs in hypocotyls and 37% (41) of the genes in cotyledons under light had opposite regulation by HFR1 and HY5 (Light, **Supplementary Figure 9**). Under shade, only 8% (29) of DEGs in hypocotyls and less than 1% (2) in cotyledons displayed opposite expression patterns in the *hfr1-5* and *hy5-215* (Shade, **Supplementary Figure 9**). This supports the cooperative roles of HFR1 and HY5 as negative regulators of SAR. To identify direct targets of this HFR1-HY5 module, shade-responsive DEGs (light vs shade, **Supplementary Figure 6**) were compiled from hypocotyls of each genotype (Col-0, *hfr1-5*, *hy5-215* and *hfr1hy5*). These hypocotyl-specific shade responsive DEGs were then compared with HY5-target genes, which were previously identified via ChIP-seq (Burko et al., 2020, **Figure 4A**). This comparison elucidated 2280 up-regulated and 2143 down-regulated hypocotyl DEGs that are HY5 targets (**Figure 4A**). The individual groups of DEGs then underwent K-means clustering of their expression fold-change to obtain the expression profile trends for these DEGs (**Figures 4B, C**). For shade up-regulated DEGs, we focused on clusters that displayed increasing trend in the fold-change (Col-0 ≤ *hfr1-5* ≤ *hy5-215* ≤ *hfr1hy5*), as these clusters represented DEGs that were down-regulated by HFR1-HY5 module under shade. As for down-regulated DEGs, we selected clusters displaying a downwards trend (Col-0 ≥ *hfr1-5* ≥ *hy5-215* ≥ *hfr1hy5*), which might be up-regulated by HFR1-HY5 module. The filtered DEGs subsequently underwent GO analysis to identify biological functions that were regulated by HFR1 and HY5 (**Figures 4D, E**).

For the up-regulated hypocotyl DEGs, gene clusters 1, 2, 5, 8 and 9 displayed upward expression trends (**Figure 4B**). Similarly, clusters 3, 6 and 8 displayed downward expression trends in the hypocotyl down-regulated genes (**Figure 4C**). Amongst the enriched GOs terms, there were many cell elongation-related GO terms such as ‘plant epidermis development’, ‘plant-type cell wall biogenesis’ and ‘unidimensional cell growth’ (**Figure 4D**). These GO terms encompass several cell wall modification genes, including *XYLOGLUCAN ENDOTRANSGLUCOSYLASE/HYDROLASE 8* (*XTH8*) and *expansin‐like A2* (*EXLA2*; **Supplementary Table 2**). Response to JA and ABA were also found to be enriched (**Figure 4D**). As for down-regulated gene clusters, the GO term related to light response such as ‘response to light stimulus’ and ‘response to light intensity’ as well as photosynthesis were enriched (**Figure 4E**; **Supplementary Table 3**).

A similar filtering process was performed for cotyledon DEGs, which resulted in 1893 up-regulated and 1879 down-regulated DEGs (**Supplementary Figure 10A**). Further clustering of the DEGs revealed clusters 1, 2, 4 and 5 showing an upwards expression fold-change from Col-0 to *hfr1hy5* for up-regulated DEGs (**Supplementary Figure 10B**). On the other hand, clusters 2, 5 and 9 (**Supplementary Figure 10C**) displayed downward trends in expression fold-change in the down-regulated cotyledon DEGs. Top enriched biological processes in up-regulated DEGs were related to abiotic and biotic stresses such as ‘response to wounding’, ‘response to heat’ and ‘response to oxidative stress’ (**Supplementary Figure 11A**; **Supplementary Table 4**). For down-regulated cotyledon DEGs, the enriched GO terms were similar to the down-regulated hypocotyl DEGs (**Supplementary Figure 11B**; **Supplementary Table 5**). This suggests that HFR1 and HY5 promote the expression of genes related to light responses and chlorophyll biosynthesis while suppressing cell elongation-related genes in the hypocotyl.

### 3.5 Organ-specific regulation of cell wall-related gene families by HFR1-HY5 module

The transcriptomic analyses of the hypocotyls have identified several GO terms related to cell elongation shared between *hy5* and *hfr1hy5* (**Figure 3**), which may contribute to the additive hypocotyl elongation observed in *hfr1hy5* double mutant (**Figure 1**). Therefore, we investigated the specific genes involved in hypocotyl elongation that were regulated by HFR1 and HY5. Several gene families related to cell wall including *XTHs*, *EXPANSINs* (*EXPs*), *Arabinogalactan proteins* (*AGPs*) and *Class III peroxidases* (*PRXs*) were enriched GO terms (**Supplementary Figure 12**). XTHs are responsible for digesting xyloglucan chains into shorter chains (Fry et al., 1992) while EXPs break the bonds between the xyloglucan chains and the cellulose microfibrils (Cosgrove, 2000), allowing the cellulose to slide across each other for cell elongation. AGPs are linked to cell expansion through their influence on cellulose biosynthesis, potentially through receptor-mediated signaling pathways (Seifert, 2018). PRXs also contribute to cell expansion by generating hydroxyl radicals from H_2_O_2_ to cleave cell wall polysaccharides (Schweikert et al., 2000; Passardi et al., 2005). Hierarchical clustering heatmaps of DEGs related to cell wall modification revealed that many DEGs from these gene families showed relatively higher expression in hypocotyls of *hfr1hy5* under light, as compared to *hfr1*-5 or *hy5-215* (Hypocotyl, **Supplementary Figure 12**). However, the expression of cell wall-related DEGs in the cotyledon did not display any obvious trends in relation to HFR1 and/or HY5 (Cotyledon, **Supplementary Figure 12**).

**Figure 5:**
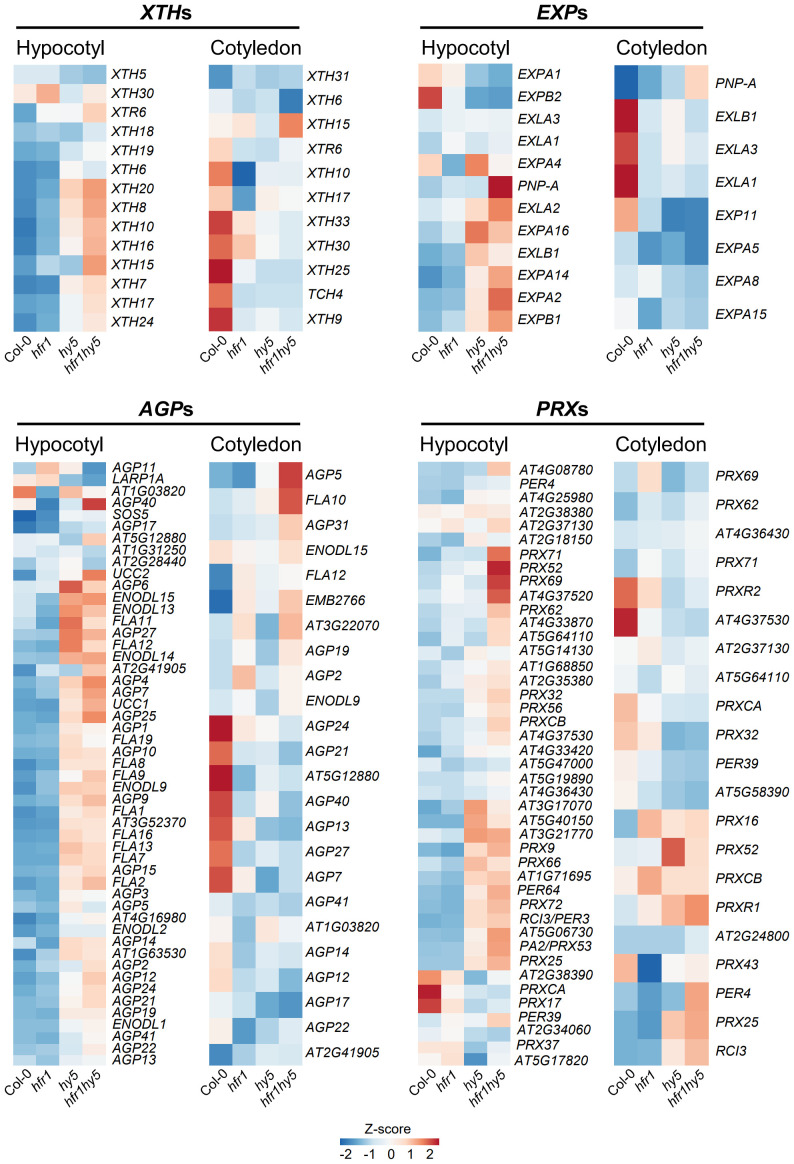
Expression analysis of cell wall-related gene families in hypocotyls and cotyledons under shade. Heatmaps displaying the expressions of cell wall-related DEGs in 1 h shade-treated Col-0, *hfr1*, *hy5* and *hfr1hy5* hypocotyls and cotyledons. *XYLOGLUCAN ENDOTRANSGLUCOSYLASE/HYDROLASEs*, *XTH*s; *EXPANSINs*, *EXPs; Arabinogalactan proteins*, *AGPs*; *Class III peroxidases*, *PRX*s.

Further analysis of these cell wall-related DEGs under shade also showed co-regulation by HFR1 and HY5 in hypocotyls (Hypocotyl, **Figure 5**). Interestingly, for cotyledon samples, many genes in *XTH*, *EXP*, *AGP* and *PRX* families showed higher expression in Col-0, which was opposite to what was observed in the hypocotyl (Cotyledon, **Figure 5**). This expression pattern correlates with the reduction of cotyledon area in *hfr1*-5, *hy5-215* and *hfr1hy5* under shade (**Figure 1F**). Overall, expression analysis of the cell wall modifying enzymes *XTH*, *EXP*, *AGP* and *PRX* gene families correlated with the elongated hypocotyl and reduced cotyledon size phenotypes observed in *hfr1*-5, *hy5-215* or *hfr1hy5*, thereby supporting the notion that HFR1 cooperates with HY5 to regulate differently the expression of these gene families in an organ-specific manner.

### 3.6 HFR1 and HY5 regulate hypocotyl responsiveness to shade through auxin metabolism

**Figure 6:**
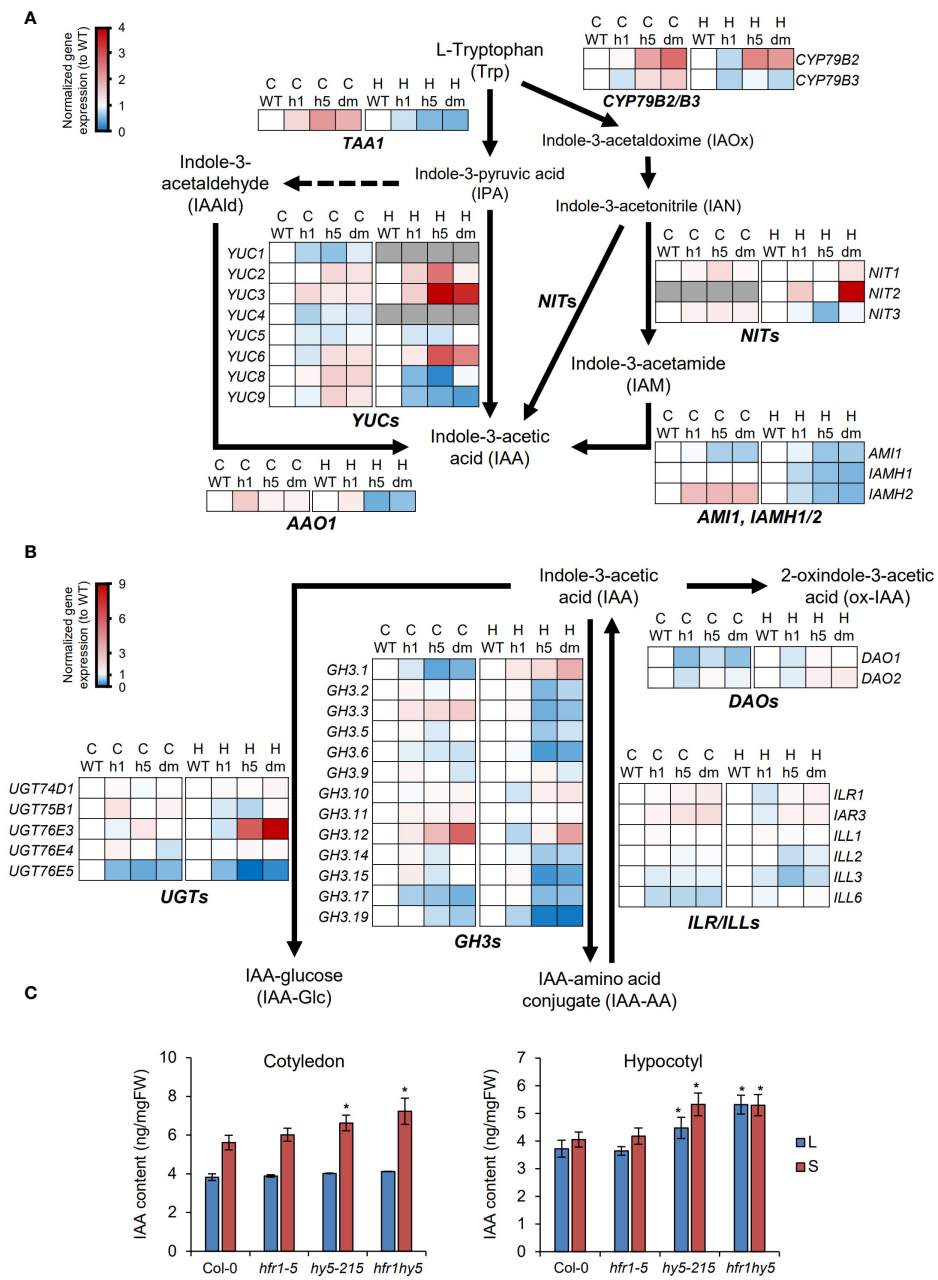
Regulation of auxin metabolism by HFR1 and HY5 in cotyledons. Expression pattern of **(A)** auxin biosynthesis genes and **(B)** auxin conjugation genes under shade in Col-0 (WT), *hfr1* (h1), *hy5* (h5) and *hfr1hy5* (dm) cotyledons (C) and hypocotyls (H). Each row represents the expression pattern of a gene in the pathway. Red and blue color represent up- and down-regulated gene expressions in comparison to WT. Arrows indicate direction of the biosynthesis pathway. *TRYPTOPHAN AMINOTRANSFERASE OF ARABIDOPSIS 1*, *TAA1*; *Cytochrome P450*, *CYP79B2/B3*; *NITRILASE* genes, *NIT*s; *ARABIDOPSIS ALDEHYDE OXIDASE 1, AAO1*; *YUCCA* genes, *YUC*s; *AMIDASE 1*, *AMI1*; *IAMHYDROLASE* genes, *IAMH1/2; GRETCHEN HAGEN 3* genes, *GH3*s; *IAA-Leu-Resistant* genes, *ILR*s; *ILR1-like* genes, *ILLs*; *UDP-glycosyltransferases*, *UGT*s; *DIOXYGENASE FOR AUXIN OXIDATION* genes, *DAO*s. **(C)** Quantification of IAA content extracted from cotyledons and hypocotyls of 7-d-old Col-0, *hfr1-5*, *hy5-215* and *hfr1hy5* seedlings grown in light (L) or shade (S). Student’s *t*-test was performed to determine significant differences compared to Col-0, within the same treatment group; *P < 0.05.

Although the expression of cell elongation genes could be directly regulated by HFR1 and HY5, these genes can also be influenced by auxin treatment (Majda and Robert, 2018). Hence, we also further explored how HFR1 and HY5 regulate auxin by observing the expression pattern of the genes involved in auxin biosynthesis pathway. The expression of auxin biosynthesis genes in both cotyledon and hypocotyl were plotted into hierarchical clustering heatmaps, displaying the relative expression levels in both light (**Supplementary Figure 13A**) and shade-treated (**Supplementary Figure 13B**) samples separately. Based on the heatmaps, two distinct groups of genes were differentially expressed in the cotyledons and hypocotyls for both light and shade-treated samples (**Supplementary Figure 13**). Under light, *TRYPTOPHAN AMINOTRANSFERASE OF ARABIDOPSIS 1* (*TAA1*), *NITRILASE 1* (*NIT1*), *NIT3*, *YUCCA* genes (*YUC1/2/3/4/5/7/8*), *AMIDASE 1* (*AMI1*), *IAMHYDROLASE1* (*IAMH1*) and *IAMH2* were highly expressed in the cotyledons, as compared to the hypocotyls (**Supplementary Figure 13A**). The other group of genes consisted of genes such as *ARABIDOPSIS ALDEHYDE OXIDASE 1* (*AAO1*), *CYTOCHROME P450 79B2* (*CYP79B2*), *CYP79B3*, *NIT2*, *YUC6* and *YUC9*, which were highly expressed in hypocotyls but not in cotyledons (**Supplementary Figure 13A**). However, under shade, *YUC9* was highly expressed in the cotyledons compared to hypocotyls while *YUC3* had higher expression in the hypocotyls (**Supplementary Figure 13B**). Further analysis of the gene expression in the auxin biosynthesis pathway revealed that under shade, genes highly expressed in cotyledons such as *TAA1*, *YUC2*, *YUC8*, *YUC9* and *IAMH2* were more up-regulated in the *hy5* and *hfr1hy5* cotyledons, compared to Col-0 (**Figure 6A**). These genes may be down-regulated by HFR1 and HY5 to modulate shade-induced IAA biosynthesis in cotyledons. For genes with high expression in hypocotyls, the expression of *CYP79B2*, *NIT2*, *YUC3* and *YUC6* were higher in *hy5* and *hfr1hy5* hypocotyls when compared with Col-0 (**Figure 6A**). This suggests that HFR1 and HY5 also suppress auxin biosynthesis in hypocotyls.

Besides auxin biosynthesis, auxin conjugation and degradation are also influenced by shade and are important in regulating free auxin required for SAR (Iglesias et al., 2018). *GRETCHEN HAGEN 3.1* (*GH3.1*), *GH3.2*, *GH3.3* and *GH3.5*, as well as *IAA-Leu-Resistant1* (*ILR1*)*-like 6* (*ILL6*) and *UDP-glycosyltransferase 76E5* (*UGT76E5*) were up-regulated in both organs under shade (**Supplementary Figure 14**). In addition, *UGT76E3* expression was also highly increased under shade though it was only observed in the cotyledons (Cotyledon, **Supplementary Figure 14**). On the other hand, *GH3.9*, *GH3.10*, *UGT74D1* and *DIOXYGENASE FOR AUXIN OXIDATION 2* (*DAO2*) were down-regulated in both organs under shade (**Supplementary Figure 14**). To deduce the influence of HFR1 and HY5 on auxin conjugation under shade, the gene expressions in *hfr1*, *hy5* and *hfr1hy5* under shade were normalized to Col-0. *GH3.1*, *GH3.3*, *GH3.12*, and *UGT76E3* expression levels were high in *hfr1hy5* compared to *hfr1* and *hy5* in either or both organs (**Figure 6B**). However, the expression of many more genes in the GH3 family (*GH3.1, 2, 3, 5, 6, 14, 15, 17* and *19*) as well as *UGT76E5* and *DAO1* were highly reduced in *hy5* and *hfr1hy5* (**Figure 6B**). This suggests that HFR1 and HY5 function to promote the expression of most auxin-conjugation genes, which may result in the reduction of free IAA under shade. Interestingly, the regulation of *GH3.1* and *GH3.3* by HFR1 and HY5 was reversed when compared between the cotyledons and hypocotyls, showing organ specificity (**Figure 6B**).

To determine the influence of gene expression on the IAA levels, the IAA levels were measured in both cotyledons and hypocotyls. Under light, the IAA levels were similar in the cotyledons of all genotypes (**Figure 6C**). However, in hypocotyls under light, IAA levels were elevated in *hy5-215*, as compared to Col-0 and *hfr1-5*, while *hfr1hy5* has an even higher accumulation of IAA than *hy5-215*, which is consistent with hypocotyls length (**Figure 6C**). This was similarly observed in cotyledons under shade, in which the increases in IAA levels were higher in *hy5-215* and compared to Col-0 and more accumulated in *hfr1hy5* (**Figure 6C**). In contrast, the hypocotyl samples showed minimal increase in IAA levels after 1 h shade treatment, with no change observed in *hfr1hy5* suggesting that IAA levels in *hfr1hy5* were saturated under light (**Figure 6C**). Taken together, this shows that HFR1 and HY5 regulate auxin biosynthesis and conjugation independently in both cotyledon and hypocotyl to regulate free IAA homeostasis under shade.

**Figure 7:**
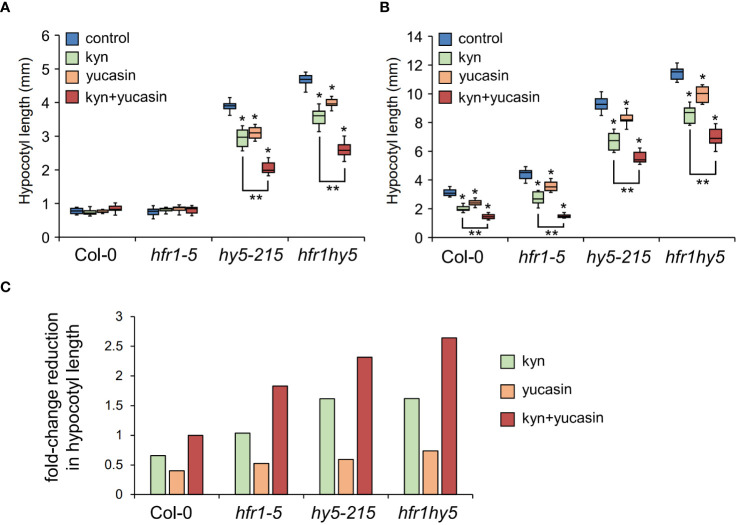
Influence of HFR1 and HY5 on hypocotyl elongation under auxin biosynthesis inhibition. Hypocotyl length of Col-0, *hfr1-5*, *hy5-215* and *hfr1hy5* mutants grown without treatment (control), with L-kynurenine (kyn), yucasin and both kyn and yucasin (kyn+yucasin) under **(A)** light and **(B)** shade condition, n=30. Welch’s *t* test was performed to determine significant differences compared to Col-0, within the same genotype (*P < 0.05) and across sample groups (**P < 0.05). **(C)** Fold-change reduction of hypocotyl length from kyn, yucasin and kyn+yucasin to control under shade in Col-0, *hfr1-5*, *hy5-215* and *hfr1hy5* seedlings. Fold-change is normalized to hypocotyl reduction with kyn+yucasin treatment in Col-0.

To verify the suppression of auxin biosynthesis by HFR1 and HY5 under shade, Col-0, *hfr1-5*, *hy5-215*, *hfr1hy5* were subjected to auxin biosynthesis inhibition using L-kynurenine (kyn), a TAA1 inhibitor, and yucasin, a YUC enzyme inhibitor. When grown under light, only *hy5-215* and *hfr1hy5* showed decreased hypocotyl length with the addition of kyn or yucasin (**Figure 7A**). Treatment with both kyn and yucasin resulted in further reduction in hypocotyl length in both *hy5-215* and *hfr1hy5* (**Figure 7A**). Under shade, addition of kyn or yucasin reduced the shade-induced hypocotyl elongation across all genotypes (**Figure 7B**). Suppression of the entire indole-3 pyruvate (IPA) pathway with both kyn and yucasin led to further decrease in hypocotyl elongation, although *hfr1hy5* still had the highest hypocotyl length (**Figure 7B**). Looking at the reduction in hypocotyl elongation with different auxin inhibitors, inhibition of *YUC* activity resulted in lower hypocotyl length reduction compared to kyn treatment (**Figure 7C**). This supports the importance of TAA1 in auxin biosynthesis, upstream of YUC in the IPA pathway (**Figure 6A**). In addition, *hfr1hy5* had the highest reduction in hypocotyl elongation under kyn+yucasin treatment, 2.5-fold higher than observed in Col-0 (**Figure 7C**). This supports the role of HFR1 and HY5 in suppressing shade-induced hypocotyl elongation through regulation of auxin biosynthesis.

## 4 Discussion

HFR1 and HY5 are well-known positive regulators of photomorphogenesis. However, they have very different modes of action. While HY5 is a typical transcription factor that associates with the promoters of its target genes to regulate their expression levels (Gangappa and Botto, 2016; Xiao et al., 2022), HFR1 is an atypical transcription factor that influences gene expression mostly by suppression of PIF activity (Pham et al., 2018; Sessa et al., 2018). Thus, the physical interaction between HFR1 and HY5 may serve as an additional layer of gene regulation. Interestingly an additive phenotype under shade was recorded in *hfr1hy5* (**Figure 1A**). Differential expression of genes is important in understanding the development of different organs (Klepikova and Penin, 2019). Hence, we separately examined the hypocotyls and cotyledons of *hfr1-5*, *hy5-215* and *hfr1hy5* under light and shade to dissect the relationship of HFR1 and HY5 in these organs.

Initial examination of the hypocotyls and cotyledons DEGs revealed higher number of DEGs regulated in hypocotyls compared to cotyledons (**Figures 2A, B**). This was similarly observed in previous studies, especially when hypocotyl growth was significantly enhanced under shade (Kohnen et al., 2016; Gao et al., 2022). Further analysis of HFR1 and HY5-regulated DEGs in hypocotyls revealed similarities in both light and shade condition (**Figure 2B**). Furthermore, the hypocotyl GO terms enriched under light and shade were also alike (**Figure 3**). This is likely due to the protein stability of HFR1 and HY5 under shade. HFR1 is very unstable by COP1-mediated degradation process under FR exposure (Jang et al., 2005). HY5 protein levels are also reduced under end-of-day FR (EOD-FR) pulse treatment (Toledo-Ortiz et al., 2014). Thus, both HFR1 and HY5 may undergo degradation under shade exposure. This could explain the increase in percentage of shared DEGs between *hy5* and *hfr1hy5* under shade (Hypocotyl, **Figure 2D**), which may result from HFR1 degradation in *hy5*. However, unlike the hypocotyls, regulation of HFR1 and HY5 on cotyledon genes is more prominent under shade (Cotyledon, **Figure 3**). This serves to highlight the difference in gene regulation by HFR1 and HY5 in different organs. HY5 is a mobile transcription factor that can travel from shoot to root (Chen et al., 2016). In addition, HY5 is also spatially regulated during thermomorphogenesis (Lee et al., 2021). The spatial regulation of HY5 may impart the ability to suppress organ-specific SAR. Besides, it is interesting to note that a large portion of the DEGs is represented by *hfr1hy5*-specifc DEGs (Hypocotyl, **Figure 2C**). These DEGs may constitute genes where HFR1 and HY5 have overlapping regulatory functions. HFR1 is well-known for suppressing PIF function (Hornitschek et al., 2009), while HY5 competes antagonistically with PIFs through shared G-box consensus sequence for binding to their target genes (Gangappa and Kumar, 2017). Hence, the overlapping regulatory functions of HFR1 and HY5 may include their shared inhibitory effect on PIF function.

This study showed that HFR1 and HY5 cooperatively regulate many biological processes including response to hormones and chlorophyll-related GO terms (**Figure 3**). Response to JA is one of the biological processes cooperatively regulated by HFR1 and HY5 (**Figure 3**). JA is a major hormone involved in inducing plant responses against biotic stresses (Campos et al., 2014). JA typically inhibits hypocotyl elongation through JA receptor CORONATINE INSENSITIVE1 (COI1; Huang et al., 2017). JA levels in *hy5-215* and *hfr1hy5* hypocotyls were reduced supporting the increased hypocotyl lengths observed (**Supplementary Figure 8**). Although GO term for ‘response to JA’ was up-regulated in *hy5* and *hfr1hy5* hypocotyls (Hypocotyl, Pink, **Figure 3**), DEGs involved in this category include *JASMONATE ZIM-DOMAIN1* (*JAZ1*), *JAZ5* and *JAZ10*, all of which are key repressors of JA signaling (**Supplementary Table 6**; Santner and Estelle, 2007). JAZ1 and JAZ10 are known to regulate shade responses through their stability (Robson et al., 2010; Leone et al., 2014). Up-regulation of *JAZs* in *hy5-215* and *hfr1hy5* hypocotyls might affect enriched GO for response to JA which would result in highly suppressed downstream JA signaling. Besides, response to ABA was similarly up-regulated in *hy5* and *hfr1hy5* hypocotyls (Hypocotyl, Pink, **Figure 3**). ABA plays an important role in regulating abiotic stress responses (Vishwakarma et al., 2017). In addition to reduced ABA levels in *hy5-215* and *hfr1hy5* hypocotyls (**Supplementary Figure 8**), up-regulated DEGs involved in ‘response to ABA’ also encompass genes such as *ABA INSENSITIVE 5 BINDING PROTEIN 1* (*AFP1*) and *AFP3* (**Supplementary Table 7**). AFPs form antagonistic interactions with ABI5 to negatively regulate ABA signaling (Garcia et al., 2008). Hence, loss of HFR1 and HY5 would result in the up-regulation of *AFPs*, leading to suppressed ABA signaling. Taken together, HFR1 and HY5 may regulate JA- and ABA-mediated responses by suppressing downstream negative regulators of the signaling pathways. Our analyses also suggest that HFR1 and HY5 cooperatively regulate chloroplast development (Hypocotyl, **Figure 3A**). HY5 is involved in regulating chlorophyll biosynthesis (Toledo-Ortiz et al., 2014). On the other hand, PIFs function as repressors of chlorophyll biosynthesis (Martin et al., 2016). HFR1 may repress PIFs to coordinate with HY5 in promoting chlorophyll biosynthesis.

Besides JA, ABA response and chlorophyll biosynthesis, the most prominent function of HFR1 and HY5 discussed in this study would be the regulation of cell elongation through regulation auxin metabolism under shade. Shade-induced IAA in the cotyledon margins (Tao et al., 2008) is transported to the hypocotyls to promote shade-regulated hypocotyl elongation (Tao et al., 2008; Keuskamp et al., 2010). Our analyses revealed that HFR1 and HY5 suppress auxin biosynthesis both in cotyledons and hypocotyls (**Figure 6A**). This is especially for *NIT2*, where expression is highest in hypocotyls of *hfr1hy5* (**Figure 6A**). *NIT1* and *NIT2* are involved in hypocotyl elongation under high temperature (van der Woude et al., 2021). Inhibition of the TAA1-YUCCAs auxin biosynthesis pathway with kyn and yucasin did not fully suppress the hypocotyl elongation in *hy5-215* and *hfr1hy5* (**Figure 7**). This suggests that in addition to the TAA1-YUCCAs auxin biosynthesis pathway, HFR1 and HY5 also regulate shade-induced hypocotyl elongation through *NIT*-dependent pathway. On the other hand, other phytohormones such as ethylene and brassinosteroid that are known to induce hypocotyl elongation (Luccioni et al., 2002; Pierik et al., 2004) may also be involved in suppression of hypocotyl elongation by HFR1 and HY5. HFR1 and HY5 promote auxin conjugation to regulate free IAA levels (**Figure 6B**). Loss of *GH3.17* in Arabidopsis showed elevated free IAA in the hypocotyl due to loss of IAA conjugation to Glu (Zheng et al., 2016). Our study found that the expression of *GH3.17* was suppressed by HFR1 and HY5 in the hypocotyls (**Figure 6B**). This indicates that HFR1 and HY5 may influence local auxin conjugation in the hypocotyl to regulate free IAA levels and hypocotyl elongation. Besides the suppression of auxin levels by HFR1 and HY5 in both organs under shade, our analysis also revealed that HFR1 and HY5 cooperatively suppress the expression levels of cell wall-modification genes in hypocotyls but enhance their expression in cotyledons (**Figure 5**). This opposing difference in expression pattern may be due to direct regulation of the cell wall modification genes. HY5 can repress cell wall-related genes such as *XTH10*, *XTH15* and *EXPA2* (Zhao et al., 2019). PIF5 is also able to directly enhance *XTH15* expression (Hornitschek et al., 2009). Although HFR1 can suppress PIF5 to reduce *XTH15* expression in conjunction with HY5, the physical interaction between HFR1 and HY5 may serve a direct regulation of *XTH15*.

In conclusion, this study demonstrates how the interaction between HFR1 and HY5 serves as a bridge to connect their function to modulate hypocotyl elongation while balancing other shade responses. Though this study focused on the regulation of auxin homeostasis and cell elongation genes by HFR1 and HY5, the transcriptome analysis also revealed other biological processes such as responses to JA and chlorophyll biosynthesis. It will be interesting to delve into the various target genes identified to better understand the function of HFR1-HY5 module in regulating in defense, other phytohormones and photosynthesis under shade.

## Data availability statement

The original contributions presented in the study are publicly available. This data can be found at the National Center for Biotechnology Information (NCBI) using accession number PRJNA1104309.

## Author contributions

IC: Data curation, Formal analysis, Investigation, Methodology, Software, Validation, Visualization, Writing – original draft, Writing – review & editing. AC: Data curation, Investigation, Validation, Writing – original draft, Writing – review & editing. BS: Data curation, Investigation, Validation, Writing – original draft, Writing – review & editing. VK: Investigation, Validation, Writing – review & editing. I-CJ: Conceptualization, Data curation, Formal analysis, Funding acquisition, Investigation, Methodology, Project administration, Resources, Supervision, Validation, Visualization, Writing – original draft, Writing – review & editing.
