# Supplementary material for: Organ-specific transcriptional regulation by HFR1 and HY5 in response to shade in Arabidopsis
Source: Front Plant Sci. 2024 Jul 31;15:1430639. doi: 10.3389/fpls.2024.1430639 (PMC11322348; doi:10.3389/fpls.2024.1430639)
Supplement: Supplementary file 1 [file Presentation_1.pdf]

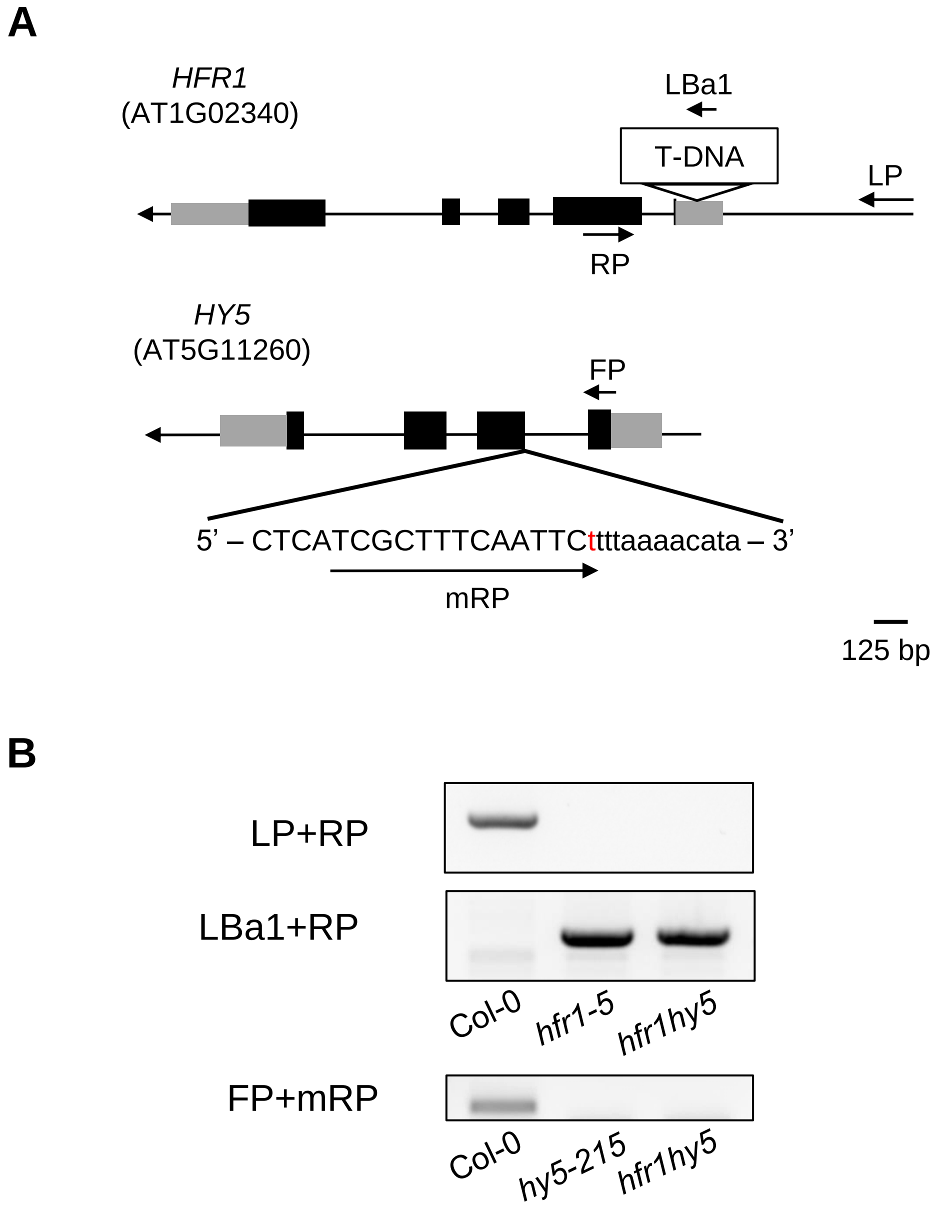

Supplementary Figure 1. PCR-genotyping of *hfr1hy5* double mutant. **(A)** Schematic representation of *HFR1* and *HY5* gene locus showing sites of T-DNA insertion in *hfr1-5* and point mutation in *hy5-215*. Black boxes and thin lines represent exons and introns respectively and grey boxes represent untranslated regions. Mutated nucleotide is labelled in red. Primers for PCR-genotyping are represented as black arrows. **(B)** PCR-genotyping of *hfr1-5*, *hy5-215* and *hfr1hy5*.

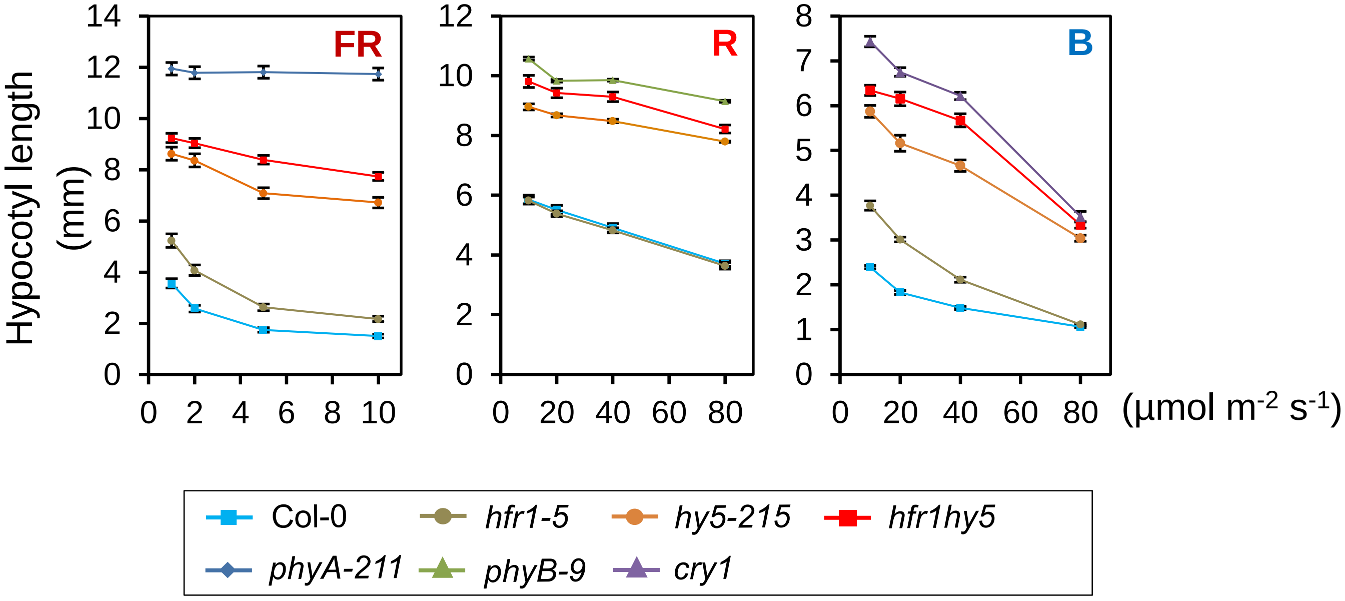

Supplementary Figure 2. Characterization of *hfr1hy5* double mutant under monochromatic light. Seedlings of Col-0, *hfr1-5*, *hy5-215*, *hfr1hy5*, *phyA-211*, *phyB-9* and *cry1* were grown in continuous far-red (FR), red (R) or blue (B) light under different intensities for 4 d. The hypocotyl lengths of the seedlings were measured and plotted against various intensities.

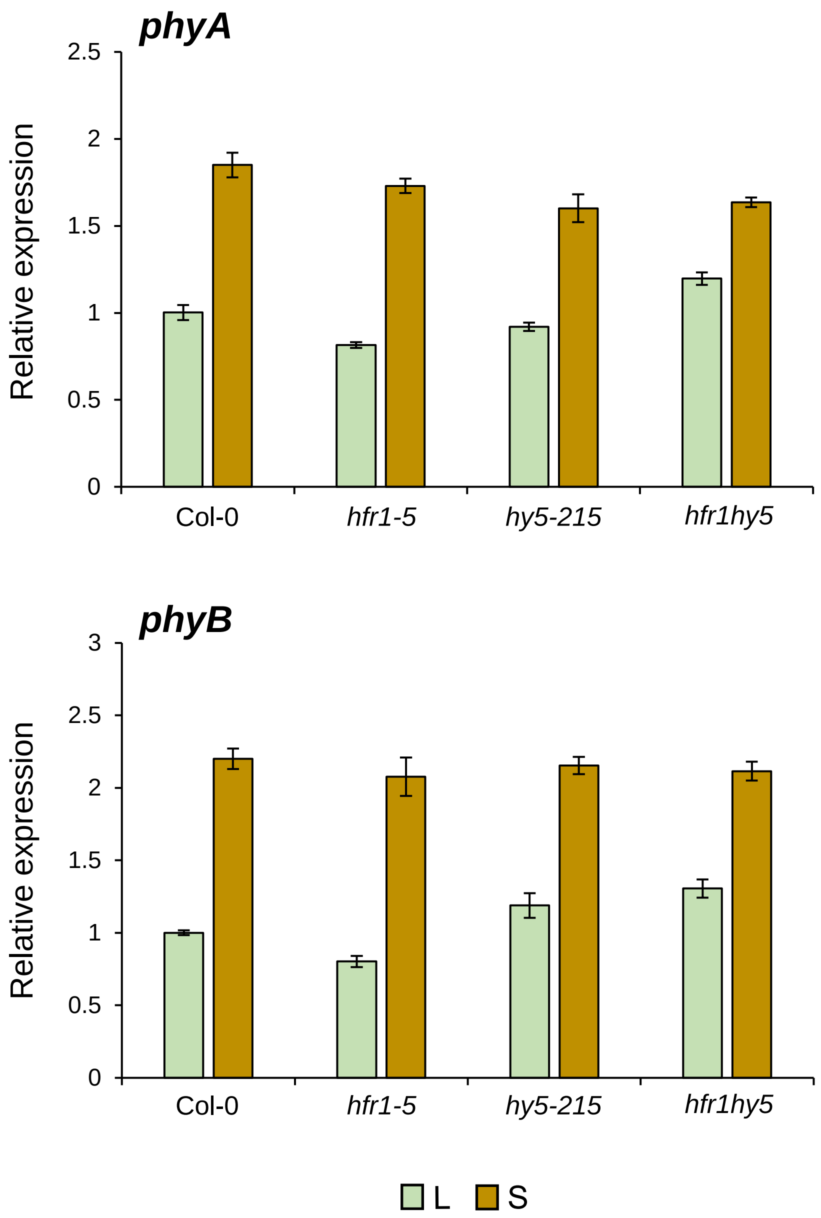

Supplementary Figure 3. Influence of HFR1 and HY5 on phytochrome expressions. qPCR of *phyA* and *phyB* expression in 7-d-old Col-0, *hfr1-5*, *hy5-215* and *hfr1hy5* seedlings under light (L) and 1 h shade (S) treatment. Student's *t*-test was performed to determine significant differences compared to Col-0, within the same treatment group: *, $P < 0.05$.

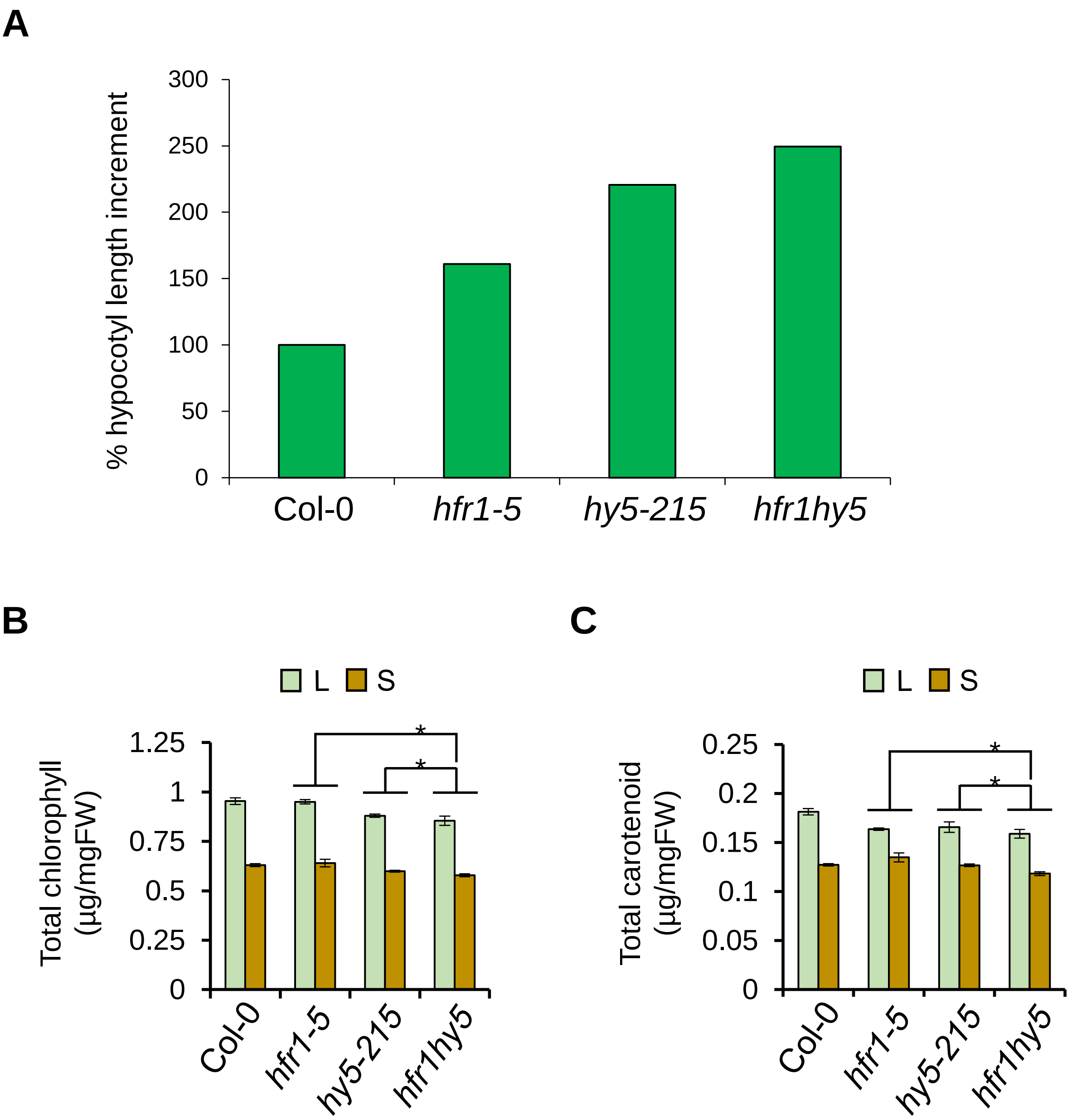

Supplementary Figure 4. Additional phenotypic characterization of *hfr1hy5* double mutant. **(A)** Percentage of hypocotyl increment from light to shade in Col-0, *hfr1-5*, *hy5-215*, *hfr1hy5* seedlings from Figure 1B. Total **(B)** chlorophyll and **(C)** carotenoid content of Col-0, *hfr1-5*, *hy5-215*, *hfr1hy5* seedlings grown under light (L) and shade (S). Student's *t*-test was performed to determine significant differences compared to Col-0, within the same treatment group; *, $P < 0.05$.

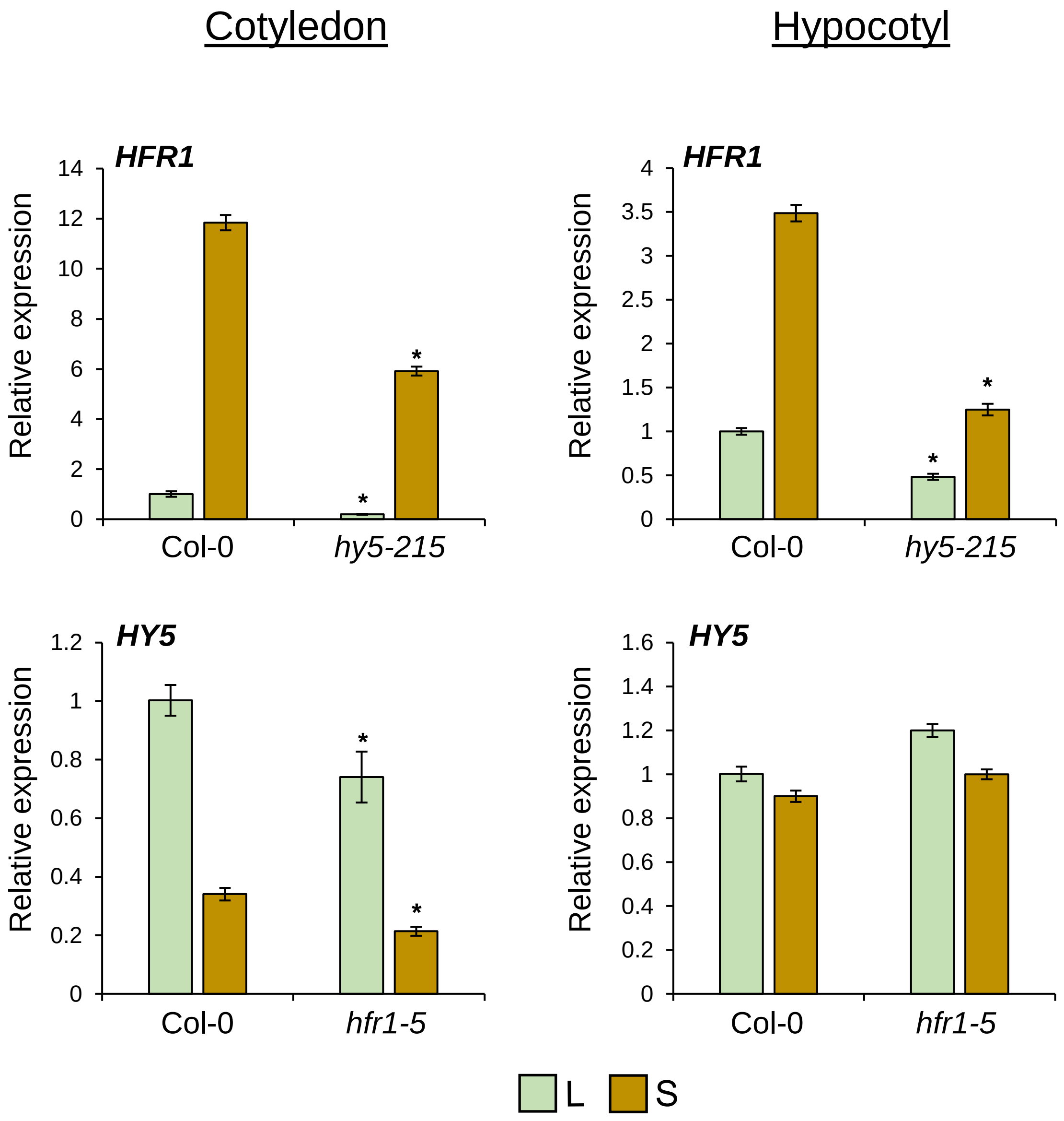

Supplementary Figure 5. Influence of HFR1 and HY5 on their individual expressions. *HFR1* and *HY5* expression in 7-d-old Col-0, *hfr1-5*, *hy5-215* seedlings under light (L) and 1 h shade (S) treatment. Student's *t*-test was performed to determine significant differences compared to Col-0, within the same treatment group; *, $P < 0.05$.

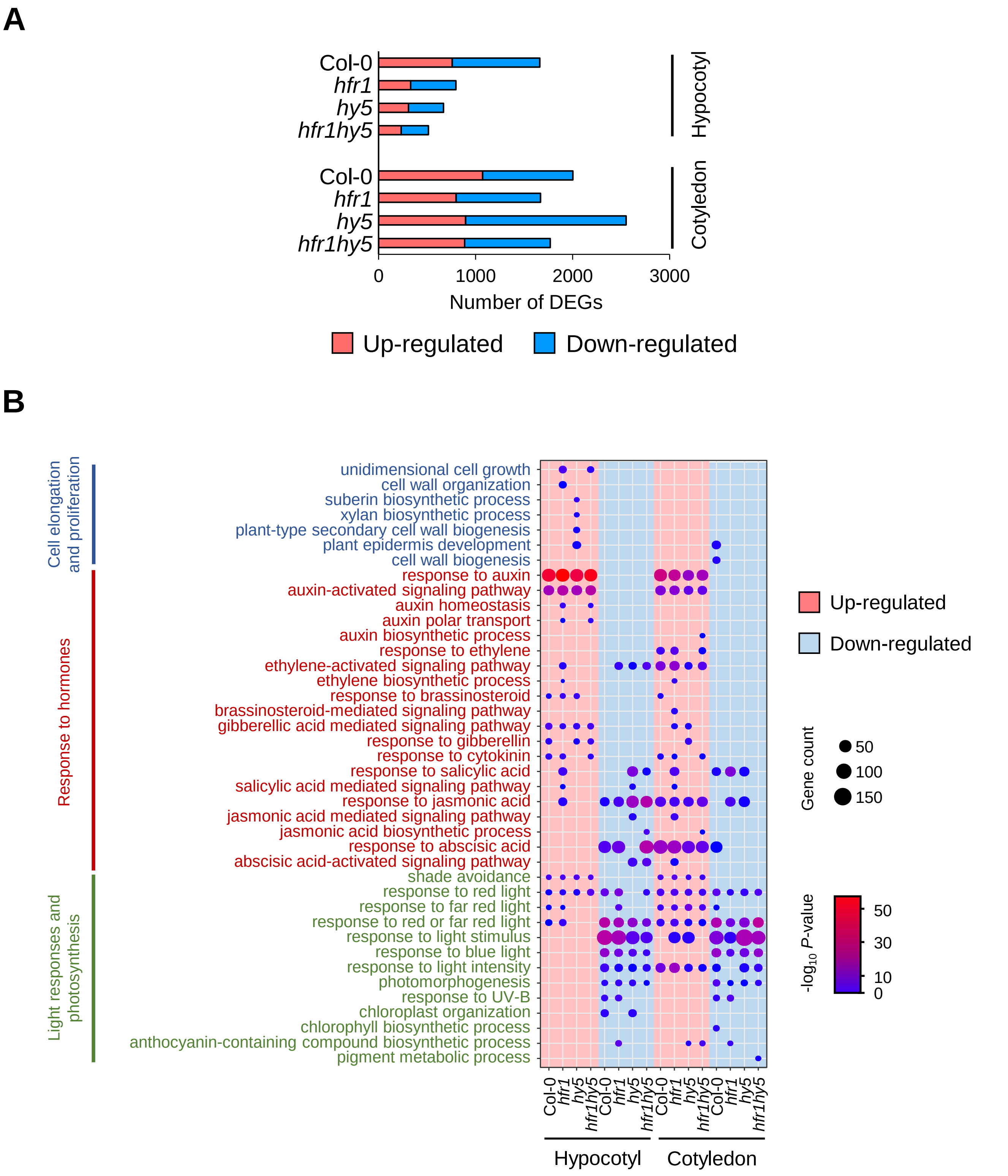

Supplementary Figure 6. DEG analysis of shade-responsive genes regulated by HFR1 and HY5. **(A)** Distribution of DEGs in 1 h shade-treated Col-0, *hfr1*, *hy5* and *hfr1hy5* hypocotyl and cotyledon samples compared to the respective light-treated genotypes. DEGs obtained from each genotype were plotted separately as up-regulated (pink, adjusted $P \leq 0.05$ and $\log_2$fold change ≥ 1), or down-regulated (blue, adjusted $P \leq 0.05$ and $\log_2$fold change ≤ -1). **(B)** Dot plot of GO terms from up- (pink) and down-regulated (blue) DEGs from (A). Dot sizes correspond to number of genes while the dot color represents the $-\log_{10}$ *P*-value.

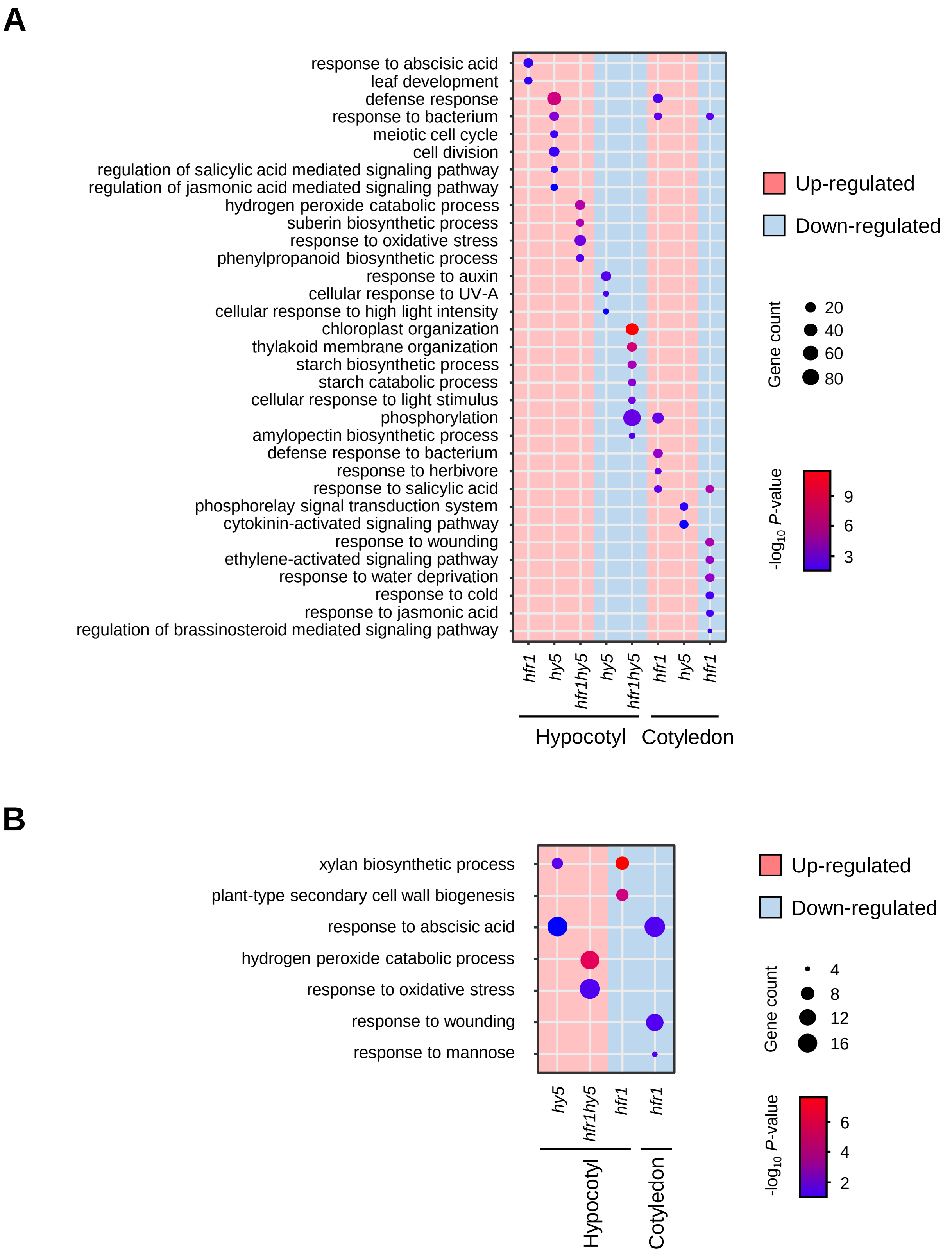

Supplementary Figure 7. GO analysis of genotype-specific DEGs under light and shade. Dot plot of GO terms enriched in up- (pink) and down-regulated (blue) DEGs from genotype-specific DEGs under **(A)** light and **(B)** shade. Dot sizes correspond to number of genes while the dot color represents the -log$_{10}$ *P*-value.

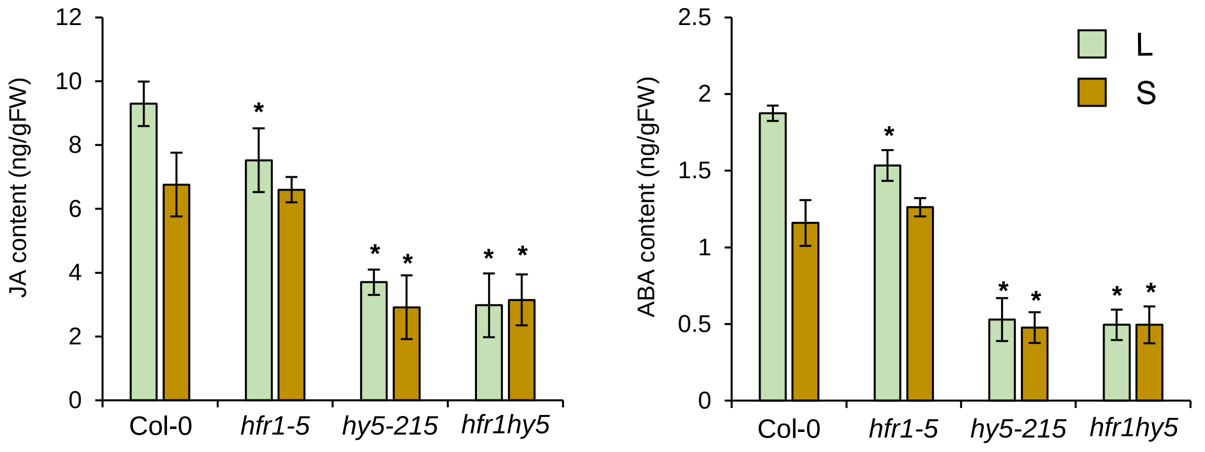

Supplementary Figure 8. JA and ABA content in hypocotyls under shade. Quantification of JA and ABA content extracted from hypocotyls of 7-d-old Col-0, *hfr1-5*, *hy5-215* and *hfr1hy5* seedlings under light (L) and 1 h shade (S). Student's *t*-tests were performed to determine significant differences compared to Col-0, within the same treatment group; *, $P < 0.05$.

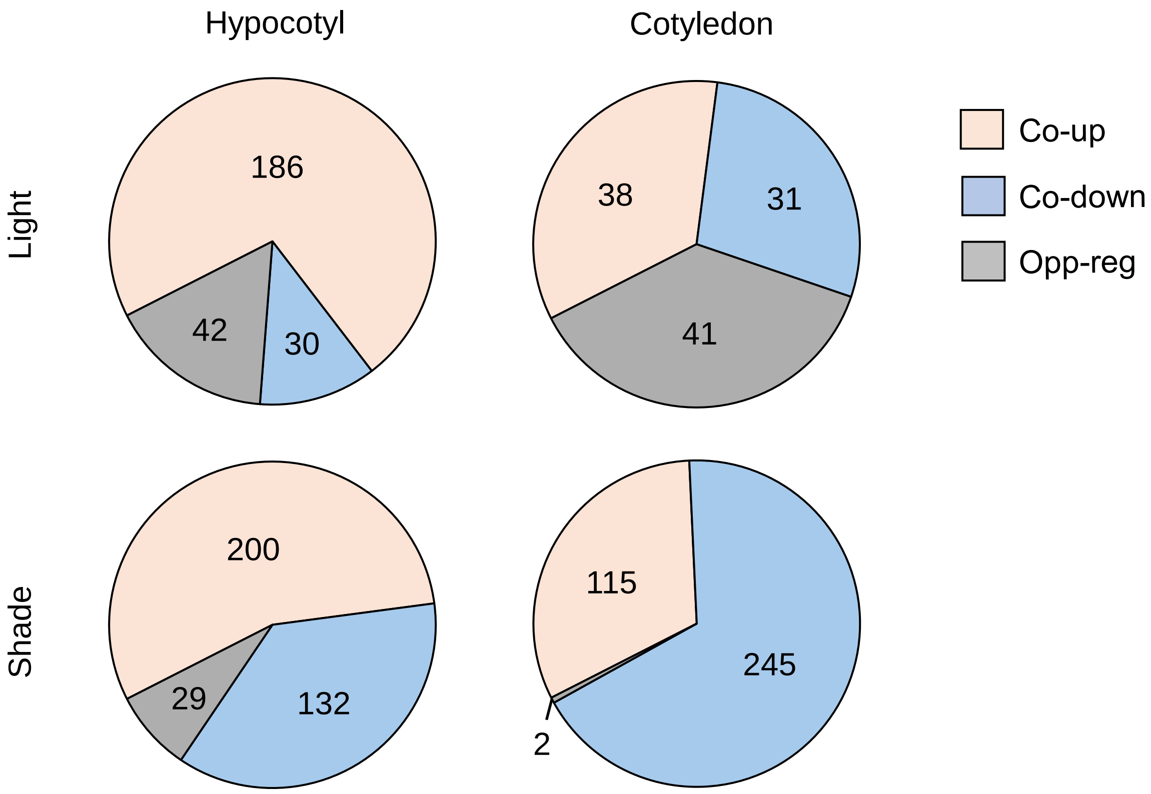

Supplementary Figure 9. Profiling of HFR1 and HY5 co-targeted genes. Pie chart of DEGs that are up-regulated (Co-up, pink) and down-regulated (Co-down, blue) by both HFR1 and HY5, and oppositely regulated (Opp-reg, grey).

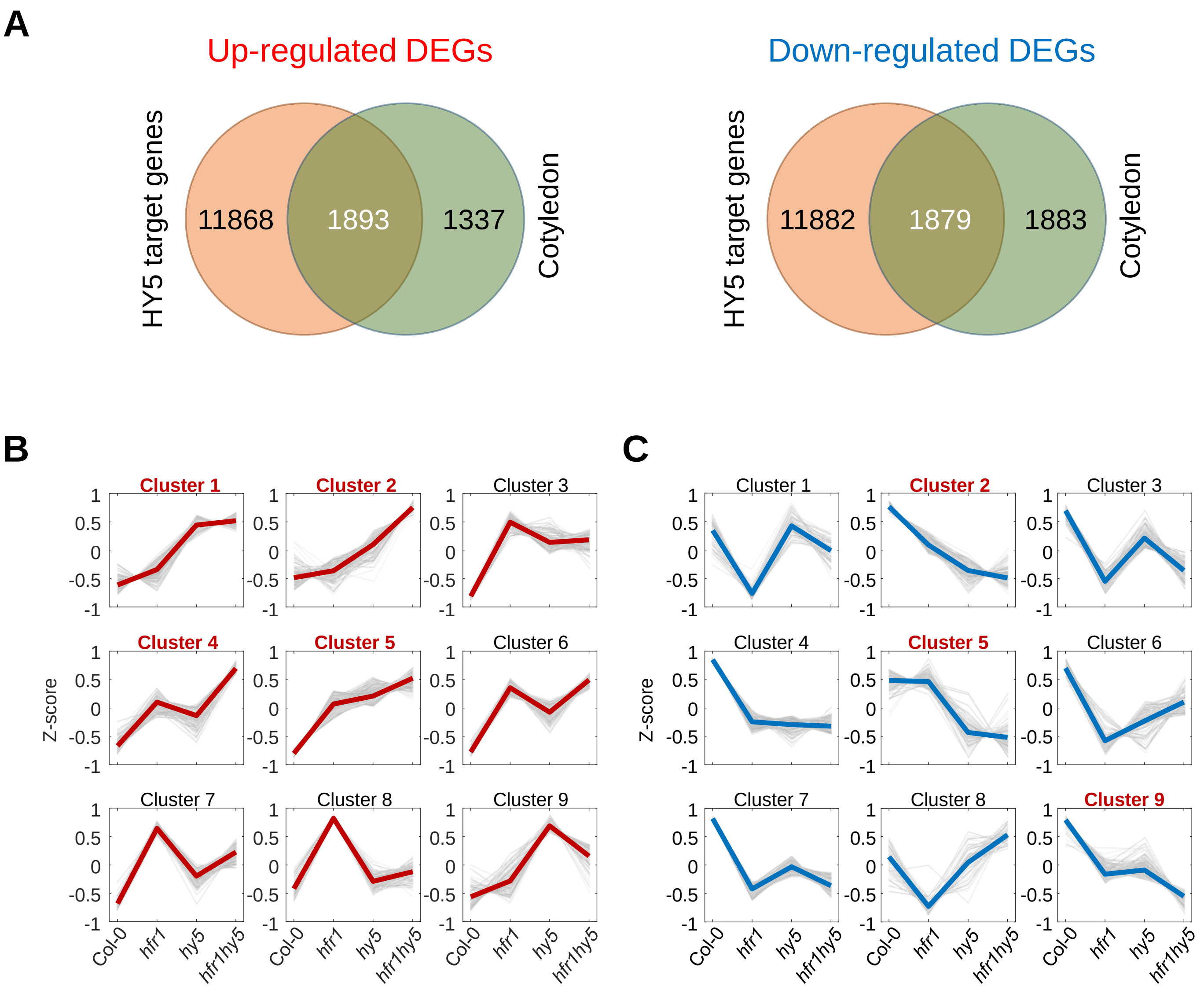

Supplementary Figure 10. Identification of DEGs targeted by HFR1 and HY5 in cotyledons under shade. **(A)** Venn diagrams display the overlap between HY5 target genes obtained from HY5 ChIP-seq data (Burko et al., 2020) and the up-regulated (left) or down-regulated (right) DEGs found in the cotyledons. Overlapped DEGs that were **(B)** up-regulated or **(C)** down-regulated under shade were categorized into nine different clusters through K-means clustering. Expression changes in y-axis represent Z-score of log_2-fold change of the DEGs. The thin grey lines represent the individual genes in each cluster. The thick lines (red or blue) represent the centroids of each clusters.

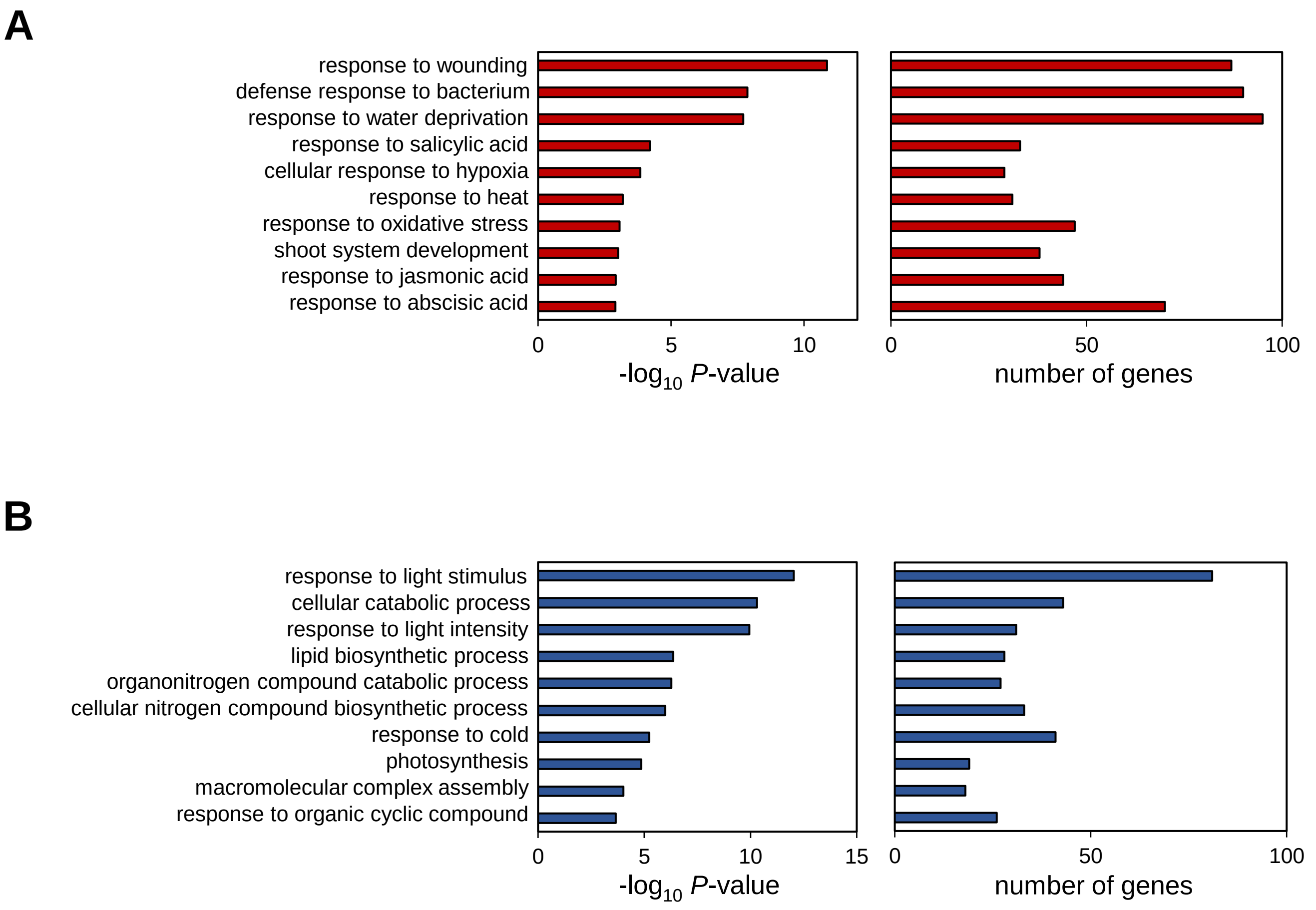

Supplementary Figure 11. Identification of biological processes co-regulated by HFR1 and HY5 in cotyledons under shade. Enriched GO terms with *P*-value ≤ 0.05 obtained from HFR1 and HY5 co-regulated genes that are **(A)** up- and **(B)** down-regulated in cotyledons from Supplementary Figure 6.

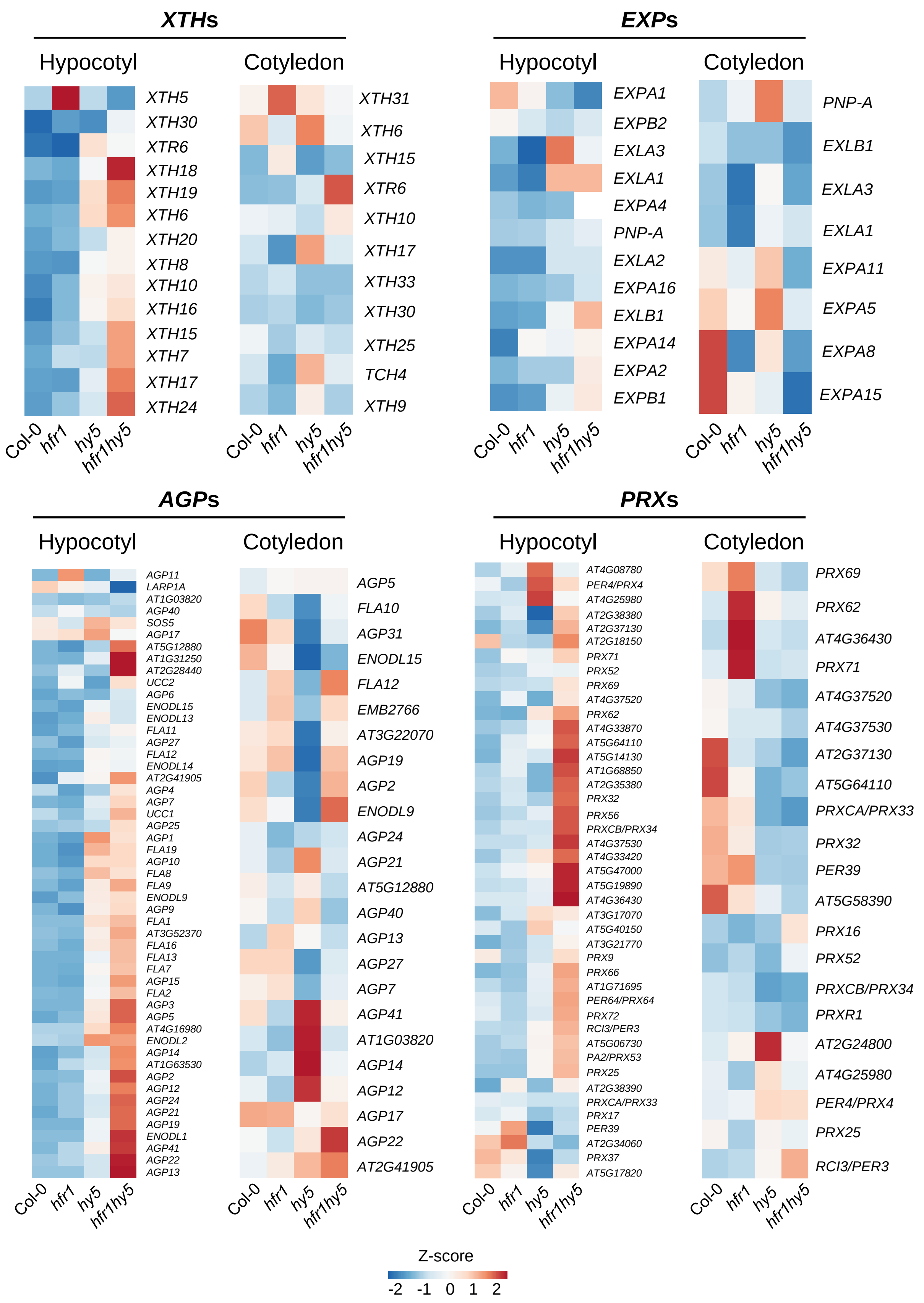

Supplementary Figure 12. Expression analysis of cell wall-related gene families in hypocotyls and cotyledons under light. Heatmaps displaying the expressions of cell wall-related DEGs in light-treated Col-0, *hfr1*, *hy5* and *hfr1hy5* hypocotyls and cotyledons. *XYLOGLUCAN ENDOTRANSGLUCOSYLASE/HYDROLASEs*, *XTHs*; *EXPANSINs*, *EXPs; Arabinogalactan proteins*, *AGPs*; *Class III peroxidases*, *PRXs*.

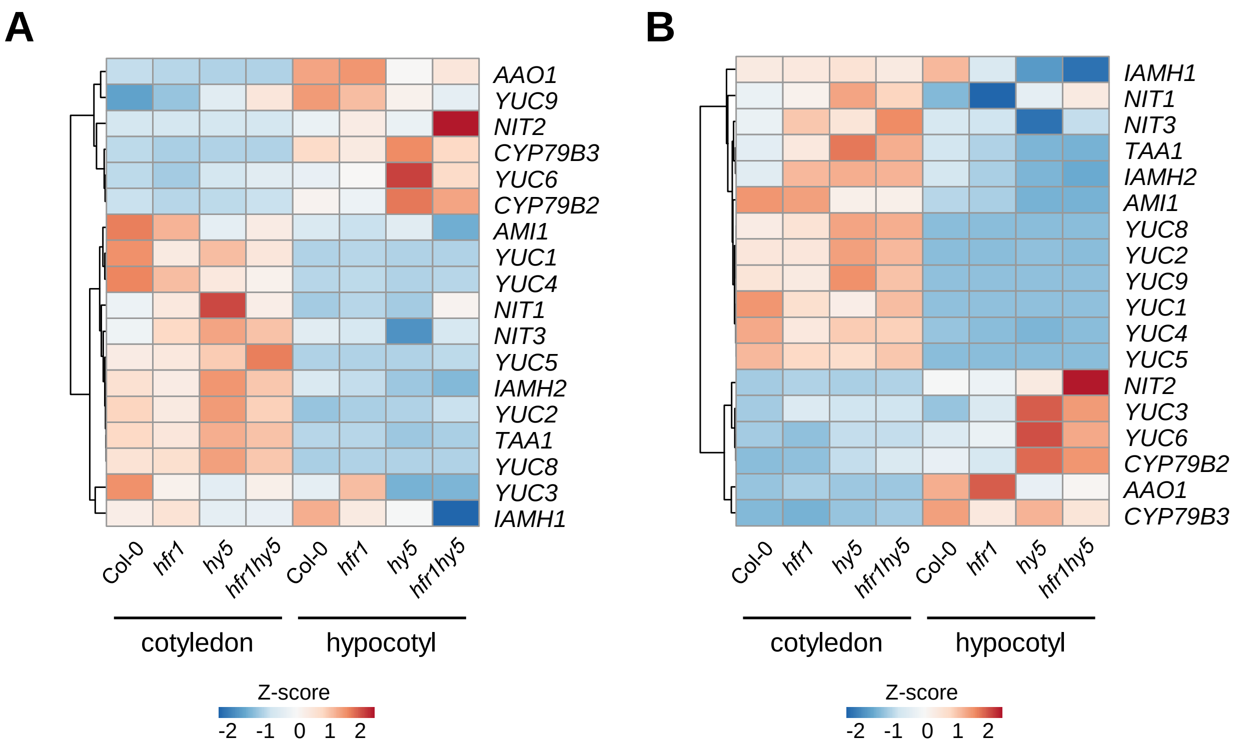

Supplementary Figure 13. Differentially expressed auxin biosynthesis genes under light and shade. Heatmap of auxin biosynthesis gene expressions under **(A)** light and **(B)** shade in Col-0, *hfr1*, *hy5* and *hfr1hy5* cotyledon and hypocotyl samples. Gene expressions are displayed as Z-scores.

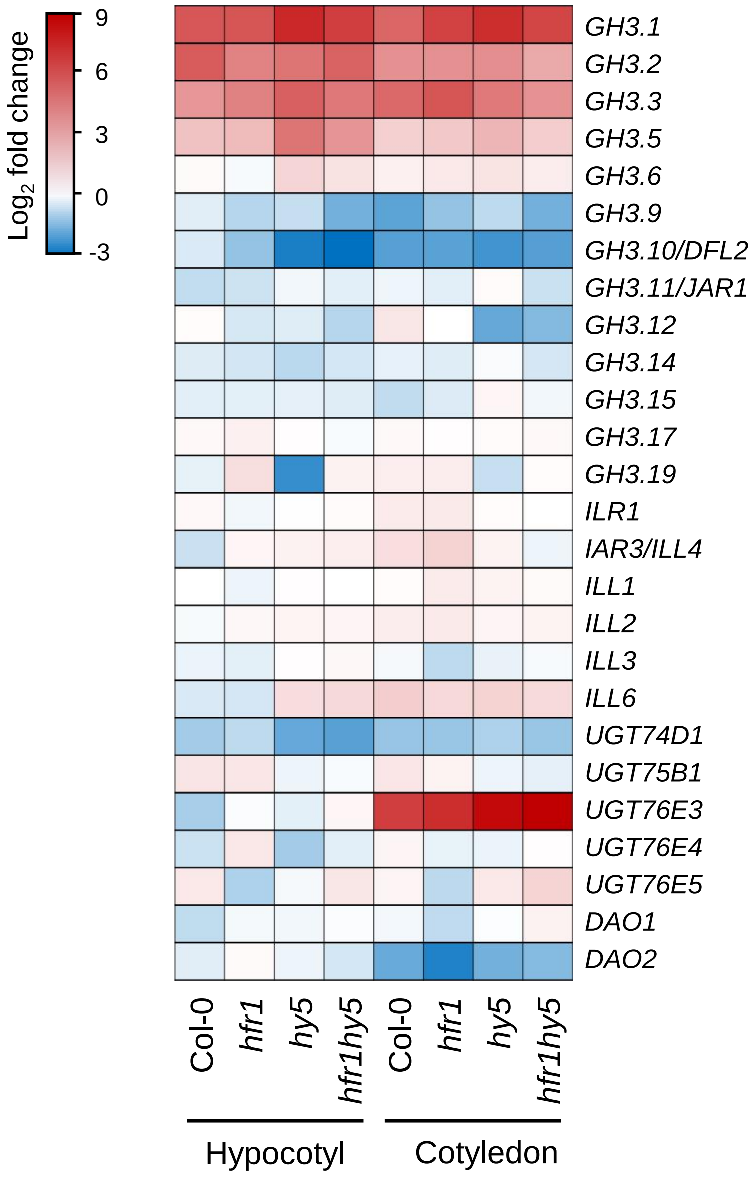

Supplementary Figure 14. Expression of auxin conjugation genes under shade. Fold change expression analysis of auxin-conjugation genes in Col-0, *hfr1*, *hy5* and *hfr1hy5* hypocotyls and cotyledons under shade in comparison to light treatment.
